# Physical Activity and Sedentary Behaviour in People with Long COVID: A Follow-Up from 12 to 18 Months After Discharge

**DOI:** 10.3390/jcm14113641

**Published:** 2025-05-22

**Authors:** Nicola S. Diciolla, Ana Ampuero-López, Alda Marques, Ana Jiménez-Martín, Sara García-De Villa, María Torres-Lacomba, María José Yuste-Sánchez

**Affiliations:** 1 Physiotherapy in Women’s Health Research Group—FPSM, Department of Nursing and Physiotherapy, University of Alcalá, Alcalá de Henares, 28805 Madrid, Spain; maria.torres@uah.es (M.T.-L.); marijo.yuste@uah.es (M.J.Y.-S.); 2 Respiratory Research and Rehabilitation Laboratory—Lab3R, School of Health Sciences—ESSUA, University of Aveiro, 3810-193 Aveiro, Portugal; amarques@ua.pt; 3 Institute of Biomedicine—iBiMED, University of Aveiro, 3810-193 Aveiro, Portugal; 4 Department of Pneumonology, University Hospital of Torrecárdenas, 04009 Almería, Spain; 5 Electronics Engineering Applied to Smart Spaces and Intelligent Transportation Systems Research Group—GEINTRA, Department of Electronics, University of Alcalá, Alcalá de Henares, 28805 Madrid, Spain; ana.jimenez@uah.es; 6 Department of Signal Theory and Communications, Rey Juan Carlos University, 28942 Madrid, Spain; sara.garcia.devilla@urjc.es; 7 Ramón y Cajal Institute of Health Research—IRYCIS, Hospital Universitario Ramón y Cajal, 28034 Madrid, Spain

**Keywords:** Long COVID, physical activity, sedentary behaviour, dyspnoea, muscle weakness, quality of life

## Abstract

**Background/Objectives**: Long-term effects of post-COVID-19 on several health outcomes remain unclear. We assessed PA and sedentary behaviour changes and explored behaviour-change factors twelve months post-COVID-19 in people with and without Long COVID. **Methods**: A prospective cohort study followed people treated for COVID-19 in different settings (home, hospital ward, intensive care unit) from twelve months to eighteen months post-COVID-19. Participants with and without Long COVID were identified. PA (Light PA-LPA, Moderate-to-Vigorous PA-MVPA, Steps·day^−1^), sedentary time, functional capacity (six-minute walk test-6MWT), muscle strength (quadriceps maximal voluntary contraction-QMVC), dyspnoea (modified Medical Research Council scale-mMRC), fatigue, symptoms of anxiety and depression, and health-related quality of life-HRQoL were assessed. **Results**: Among 148 participants (58 ± 15 years, 54% male), 101 had Long COVID. All remained physically inactive. People with Long COVID significantly increased LPA (LPA_LongCOVID_ +28 [1; 55] min·day^−1^; LPA_Controls_ +6 [−32; 45] min·day^−1^), and decreased MVPA (MVPA_LongCOVID_ −4 [−7; −2] min·day^−1^; MVPA_Controls_ −4 [−8; 1] min·day^−1^) and sedentarism (Sedentarism_LongCOVID_ −47 [−89; −4] min·day^−1^; Sedentarism_Controls_ −30 [−88; 28] min·day^−1^). At eighteen months, higher proportions of individuals with Long COVID had impaired 6MWT (17% vs. 0%), reduced QMVC (25% vs. 6%), dyspnoea (24% vs. 0%), fatigue (67% vs. 13%), symptoms of anxiety (47% vs. 9%) and depression (26% vs. 0%) as well as poor HRQoL (50% vs. 6%). PA and sedentary behaviour changes at eighteen months were associated with dyspnoea and impaired QMVC at twelve months (LPA: mMRC ≥ 2: −41.56 [−129.30; 46.00] min·day^−1^, Steps·day^−1^: mMRC: −416.13 [−1223.83; 391.57]; QMVC ≤ 70% predicted: −1251.39 [−2661.69; 158.91], Sedentarism: mMRC ≥ 2: +47.21 [−90.57; 184.99] min·day^−1^; 0.24 ≤ R^2^ ≤ 0.32). **Conclusions**: PA and sedentary behaviour remain altered long after COVID-19, with people with Long COVID adjusting to fit lower PA levels, possibly driven by physical limitations and symptoms. Dyspnoea and muscle weakness may influence PA and sedentary behaviour.

## 1. Introduction

Coronavirus disease 2019 (COVID-19) remains a major public health challenge worldwide [1]. Long COVID, also referred to as post-COVID-19 condition [2], affects an estimated 36% of people with confirmed SARS-CoV-2 infection globally, with significant variation in prevalence across populations and symptoms persistence over twelve months [3]. Its burden increases with acute disease severity, ranging from 29% of non-hospitalised people to 44% of hospitalised patients [3,4]. Although Long COVID can occur regardless of initial disease severity [3,5,6], it is more frequently reported in females than in males (45% vs. 37%), and risk of being affected is further increased in people aged 40–54 years or above 65 years and with pre-existing morbidities (e.g., obesity, cardiovascular disease) [3,7,8].

Symptom prevalence often increases with time since the acute infection. When stratified by follow-up durations of <12 months and ≥12 months, increases are observed across symptom subtypes, e.g., musculoskeletal (8% to 30%), psychological (10% to 29%), neurological (13% to 27%), general fatigue (19% to 26%) [3]. Persistent symptoms include fatigue, dyspnoea, pain, and cognitive impairment [5,9,10], and may result from residual organ damage or ongoing inflammatory responses [6,11]. These symptoms may substantially compromise physical capacity and reduce engagement in daily activities, promoting physical inactivity and impairing health-related quality of life [1,5,12]. Therefore, a downward spiral of inactivity, further symptom exacerbation and functional decline is often observed [13].

While physical inactivity health risks have been recognised, limited research has explored physical activity (PA) patterns in people after COVID-19, specifically among those with Long COVID. Available evidence suggests that non-hospitalised patients are generally more active and less sedentary than those hospitalised, three months after discharge (self-reported data: 1344 ± 911 vs. 785 ± 735 total MET/min·week^−1^; 339 ± 135 vs. 441 ± 127 sitting min·day^−1^) [14]. Nonetheless, all subgroups, whether hospitalised or not, show reduced PA at six months after discharge than before infection (self-reported data: median [Q1; Q3]: 90 [30; 150] vs. 120 [60; 240] walking min·week^−1^) [15]. Furthermore, hospitalised patients, regardless of their acute disease severity, remain largely inactive (objectively measured data: 18.9 ± 20.2 min·day^−1^, in moderate-to-vigorous PA) and sedentary (objectively measured data: 12.4 ± 1.7 h·day^−1^, in sedentary time) eight months after discharge [16]. However, few studies have objectively assessed PA and sedentary behaviour beyond twelve months after discharge, a critical period when people are expected to have resumed their daily activities but may still experience functional limitations [17]. Understanding the long-term influences of COVID-19 on PA and sedentary behaviour, as well as in other health outcomes, is crucial for designing long-term rehabilitation programmes and reducing the risks of inactivity-related complications [18]. This study aimed to assess changes in PA and sedentary behaviour and explore potential behaviour-change factors twelve months after COVID-19 in people with and without Long COVID.

## 2. Materials and Methods

### 2.1. Study Design

An observational prospective study was conducted, following a cohort of patients with SARS-CoV-2, treated in different clinical contexts (home, hospital ward, intensive care unit). All participants provided informed consent, and data protection was ensured, aligning with European regulations, maintaining anonymity by encoding files. This study was approved by the Ethics Committee for Research and Animal Experimentation, University of Alcalá (CEID/HU/2020/51), 18 December 2020, and the Ethics Committee for Drug Research of the Guadalajara Health District, Hospital Universitario de Guadalajara (CEIm/01/2021), 25 January 2021, and registered on clinicaltrials.gov (NCT04768257). It was reported following the guidelines for Strengthening the Reporting of Observational Studies in Epidemiology [19] and Transparent Reporting of a Multivariable Prediction Model for Individual Prognosis or Diagnosis [20].

### 2.2. Participants

Participants were recruited from a database of patients with COVID-19 (n = 675), who were treated and then followed at the Hospital Universitario de Guadalajara or primary care centres in Guadalajara, Spain, between March and June 2020, during the Spanish COVID-19 outbreak first wave. Participants were included if they were aged ≥ 18 years, had a COVID-19 diagnosis confirmed via polymerase chain reaction test, and were clinically stable twelve months post-hospital/medical discharge. Post-COVID-19 condition or Long COVID was diagnosed in people reporting symptoms persisting for at least two months following a laboratory-confirmed, probable, or suspected COVID-19 infection, provided symptoms had lasted for at least three months post-infection. Diagnosis was based on the World Health Organisation definition [2,12] and was determined by a pulmonologist after clinical assessment. Given the lack of universally accepted diagnostic criteria, this classification was made using available guidelines and expert judgment. People were excluded if they had severe medical conditions, including cognitive impairment or significant cardiovascular, neurological, or musculoskeletal diseases that could limit their participation. Furthermore, those vaccinated before infection or who experienced reinfection during follow-up were also excluded.

### 2.3. Procedures

Eligible participants were contacted and invited for a first visit by a pulmonologist or a respiratory physiotherapist between April and September 2021, during which those interested provided informed consent. Participants were interviewed and medical records reviewed to collect sociodemographic, anthropometric and clinical data. Then, functional capacity, peripheral muscle strength, and lung function were assessed using several tests, and dyspnoea, fatigue, anxiety, depression, and health-related quality of life (HRQoL) through self-administered questionnaires. Finally, people were provided with a specific device and instructed on its use for measuring daily PA and sedentary time over one week. A pulmonologist, specialised nurse, or technician from the corresponding centre performed and assessed spirometry, while two respiratory physiotherapists conducted and supervised all other tests in a reserved space at the same centre. The assessments were scheduled at three time points as follows: at twelve months post-hospital/medical discharge (baseline), three (fifteen months) and six months (eighteen months) afterwards.

### 2.4. Physical Activity and Sedentary Behaviour

PA and sedentary behaviour, the main outcomes, were assessed using custom-designed inertial systems (triaxial accelerometer and gyroscope). Participants wore the device on the lower back for seven consecutive days during waking hours (approx. 7.00 a.m.–10.00 p.m.), excluding water-based activities. A valid assessment required ≥8 h/day of wearing time and ≥4 valid weekdays were needed for inclusion [21]. Participants received verbal and written instructions and logged wear time interruptions. Data were processed using MATLAB (MathWorks, MATLAB (R2024a), Natick, MA, USA) to extract 31 features per three-second window (two-second overlap). PA levels were classified using a semi-supervised support vector machine (polynomial kernel) [22,23] trained on labelled data from healthy participants with activity diaries and tested on held-out data (error rate: 11%). Aggregate step counts [24] and time in PA levels (light: 1.5–2.9 METs·day^−1^; moderate-to-vigorous: ≥3.0 METs·day^−1^) and sedentary behaviour (<1.5 METs·day^−1^) [25] were averaged across valid days and adjusted for daylight hours. Inertial systems have already been shown to be valid and reliable in other clinical populations [26].

### 2.5. Secondary Outcomes

Functional capacity was assessed using the 6-min walk test/distance-6MWD following the European Respiratory Society/American Thoracic Society-ERS/ATS guidelines [27] and 1-min sit-to-stand test-1minSTS with a standardised procedure [28]. Peripheral muscle strength was evaluated through the dominant-leg quadriceps maximal voluntary contraction (QMVC), measured using a hand-held dynamometer (MicroFET2, Acme Corporation, Springfield, IL, USA) [29] according to a standardised protocol [30].

Percentages predicted were computed [31,32,33], and the proportions of people with impaired functional capacity and peripheral muscle strength (i.e., values ≤70% predicted) were determined.

Dyspnoea, fatigue and symptoms of anxiety and depression were assessed using the modified Medical Research Council-mMRC dyspnoea scale [34], the Functional Assessment of Chronic Illness Therapy-Fatigue-FACIT-FS [35], and the Hospital Anxiety and Depression Scale-HADS [36], respectively. HRQoL was evaluated using the European Quality of Life-Five dimensions-Five levels-EQ-5D-5L questionnaire [37].

The proportions of participants with clinically relevant symptoms (with values equal or higher than two on the mMRC dyspnoea scale, ≤43 on the FACIT-FS, values equal or higher than eight on the HADS subscales [34,35,36]) and impaired HRQoL (with values ≤ 74 for male and ≤78 for female on the EQ-5D-5L visual analogue scale [38]) were then reported.

Sociodemographic (age, sex), anthropometric (height, weight), and clinical characteristics were collected from structured interviews and medical records. Comorbidities were classified using the International Classification of Diseases, 11th edition [39], and the Charlson Comorbidity index [40]. Medications were categorised according to the Anatomic, Therapeutic and Chemical classification system [41]. Metabolic, cardiovascular, musculoskeletal, respiratory, immune and nervous comorbidities, along with related pharmacological treatments, were reported as being associated with impaired physical function and symptoms and, consequently, as potential modifiers of PA and sedentary behaviours [18]. Lung function was assessed through spirometry, following the ERS/ATS statement [42], with forced respiratory volume in one second and forced vital capacity percentages predicted (FEV_1_ and FVC% predicted, respectively) registered.

### 2.6. Statistical Analyses

All analyses were performed using SPSS^®^ Statistics v.29 (IBM Corp., Armonk, NY, USA) and the R package (R v.4.4.2, R Foundation, Vienna, Austria; RStudio v.2024.12.0+467, PBC, Boston, MA, USA). Quantitative data were summarised as mean ± SE, mean ± SD or median (Q1; Q3), based on the Shapiro–Wilk test, histogram and Q-Q plot inspection, and skewness/kurtosis. Categorical variables were reported as absolute and relative frequencies.

#### 2.6.1. Physical Activity and Sedentary Behaviour in People with and Without Long COVID

Sample size was estimated using G*Power software (v.3.1.9.4, Universität Kiel, Kiel, Germany). The effect size was based on patient-reported data due to the lack of prospective, objectively measured PA data in people after COVID-19 [15]. A change of −50 ± 117 min/day over six months, assuming d = 0.44, 95% power, α = 0.05, and 20% dropout rate, required 98 participants (49/group; F test: ANCOVA: repeated measures, within-between interaction).

Mixed models (restricted maximum likelihood, fixed effects, type III sum of squares) and Friedman tests were used for between-group comparisons of continuous data, with model assumptions (normality of residuals, homoscedasticity, multicollinearity) assessed accordingly. Generalised linear mixed models (restricted maximum likelihood, fixed effects, binomial distribution, logit link, robust standard error), Cochran’s Q and Fisher’s exact tests were used for categorical data. Covariance structure (compound symmetry, unstructured, autoregression) was selected using likelihood ratio tests, Akaike and Bayesian information criteria. All analyses were adjusted for age and sex. Post hoc pairwise between-group comparisons were corrected using the Bonferroni method (*p*-value < 0.017) or sequential Holm–Bonferroni procedure, with degrees of freedom estimated using the Satterthwaite method. Within-group changes were considered statistically significant at a *p*-value < 0.05.

The same statistical procedures were used for exploratory analyses comparing groups classified by acute disease severity (home-H, hospital ward-HW, intensive care unit-ICU).

#### 2.6.2. Potential Contributing Factors for PA and Sedentary Behaviour Change

Prediction models were built using PA outcome measures at eighteen months (light, moderate-to-vigorous PA, steps/day, sedentary time) as dependents, and twelve-month predictors (e.g., age, BMI, FEV_1_% predicted, FVC% predicted, 6MWD, 1 min STS, QMVC, number of symptoms, mMRC, FACIT-FS, HADS-A, HADS-D, EQ-5D-5L, PA measures). Predictor selection was based on (i) literature (e.g., dyspnoea, fatigue), (ii) group comparisons by PA trajectory (decrease/increase over six months) using unpaired *t*-tests, Mann–Whitney U, and Fisher’s exact test, and (iii) correlation analyses (Pearson, Spearman) between eighteen-month PA outcome-measures vs. twelve-month predictors.

Based on a six-month change of −864 ± 3032 steps/day (previous analysis), with f^2^ = 0.34, 95% power, α = 0.05, and 11 predictors (previously identified), 85 participants were required (F test: linear multiple regression: fixed model).

Initial models used stepwise regression (entry *p*-value < 0.05, removal *p*-value > 0.10). Multicollinearity was assessed using tolerance (<0.10) and variance inflation factor (>10.00), and confirmed with eigenvalues and condition indexes (>15.00). Least Absolute Shrinkage and Selection Operator (LASSO) regression with 10-fold cross-validation refined models, bootstrap resampling assessed internal validity [43].

Model calibration was evaluated using slope (ideal = 1) and intercept (ideal = 0); slope <1 indicated overfitting, while non-zero intercepts suggested systematic bias.

## 3. Results

### 3.1. Participants’ Baseline Characteristics

**Figure 1:**
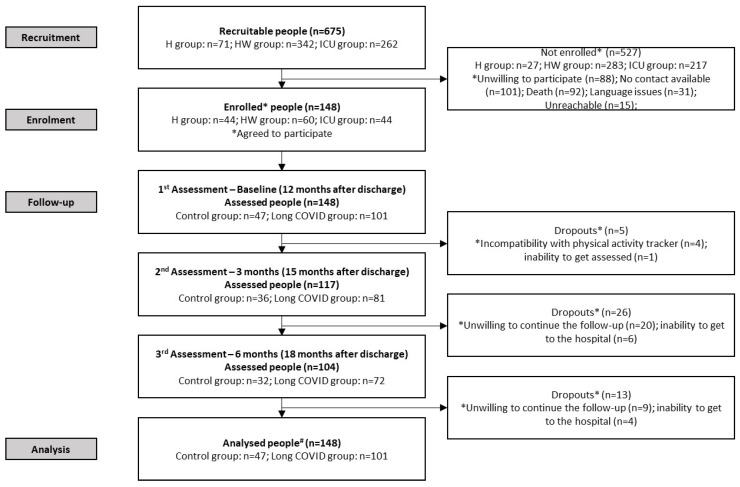
Flowchart of people with and without Long COVID, during a six-month follow-up, starting from twelve months post-discharge. Abbreviations: COVID: Coronavirus Disease 2019; H, home; HW, hospital ward; ICU, intensive care unit. ^#^ The mixed model analysis allowed for managing missing data.

One hundred and forty-eight people were recruited at twelve months after COVID-19, of whom 101 met the diagnostic criteria for Long COVID. One hundred and four completed the 6-month follow-up (Figure 1).

People with Long COVID were older (60 ± 14 vs. 54 ± 17 years), more comorbid [2 (1; 3) vs. 1 (0; 2)] and medicated [1 (1; 2) vs. 1 (0; 1)] than controls (Table 1).

### 3.2. Physical Activity and Sedentary Behaviour in People with and Without Long COVID

People with Long COVID and controls remained physically inactive throughout the follow-up period, not meeting the World Health Organisation PA recommendations (i.e., ≥150 min·week^−1^ of moderate PA, or ≥75 min·week^−1^ of vigorous PA, or an equivalent combination of both) [44].

**Figure 2:**
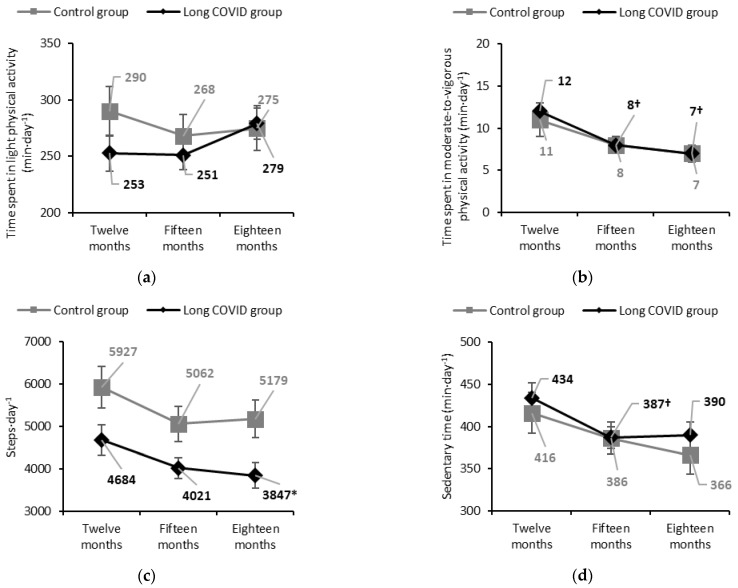
Physical activity and sedentary behaviour changes of people with and without Long COVID, during six-month follow-up, starting from twelve months post-discharge; (**a**) LPA: Control group: −24 [−73; 33] min·day^−1^, *p* = 1.00; −15 [−70; 41] min·day^−1^, *p* = 1.00; Long COVID group: −2 [−41; 37] min·day^−1^, *p* = 1.00; +25 [−14; 65] min·day^−1^, *p* = 0.35. (**b**) MVPA: Control group: −4 [−8; 1] min·day^−1^, *p* = 0.13; −4 [−8; 1] min·day^−1^, *p* = 0.08; Long COVID group: −3 [−6; −1] min·day^−1^, *p* = 0.02 †; −4 [−7; −2] min·day^−1^, *p* < 0.01 †. (**c**) Steps·day^−1^: Control group: −866 [−998; 266], *p* = 0.20; −749 [−2021; 523], *p* = 0.46; Long COVID group: −663 [−1489; 164], *p*= 0.16; −837 [-1753;79], *p* = 0.09. (**d**) Sedentary time: Control group: −30 [−88; 28] min·day^−1^, *p* = 0.63; −51 [−114; 13] min·day^−1^, *p* = 0.17; Long COVID group: −47 [−89; −4] min·day^−1^, *p* = 0.03 †; −43 [−88; 2] min·day^−1^, *p* = 0.07. Changes from twelve months are expressed as mean difference [95% CI]. † Statistically significant *p*-value < 0.05 within group. * Statistically significant *p*-value < 0.017 between groups. Abbreviations: COVID: Coronavirus Disease; LPA: time spent in light physical activity; MVPA: time spent in moderate-to-vigorous physical activity.

Among people with Long COVID, light PA significantly increased between fifteen and eighteen months (+28 [1; 55] min·day^−1^). However, moderate-to-vigorous PA significantly decreased at fifteen months (−3 [−6; −1] min·day^−1^) and this reduction was maintained at eighteen months (−4 [−7; −2] min·day^−1^). Daily step count also declined between twelve and eighteen months (−837 [−1753; 79] steps·day^−1^, ≥600 steps·day^−1^ threshold) [45], although this change was not statistically significant. Sedentary time decreased at fifteen months (−47 [−89; −4] min·day^−1^), and this reduction was sustained at eighteen months (−43 [−88; 2] min·day^−1^). The proportion of people with Long COVID walking fewer than 5000 steps·day^−1^ increased significantly from 36% at twelve months to 61% at eighteen months. In the control group, no significant changes were observed (Table 2 and Figure 2).

### 3.3. Potential Contributing Factors for Physical Activity and Sedentary Behaviour Change

PA and sedentary behaviour changes over six months were associated with dyspnoea and QMVC at twelve months. Greater dyspnoea severity was linked to lower light PA (mMRC: β = −21.16 [−61.87; 19.55] min·day^−1^; mMRC ≥ 2: β = −41.56 [−129.30; 46.00] min·day^−1^), fewer steps/day (mMRC: β = −416.13 [−1223.83; 391.57]) and higher sedentary time (mMRC: β = +24.10 [−34.60; 82.80] min·day^−1^; mMRC ≥ 2: β = +47.21 [−90.57; 184.99] min·day^−1^) at eighteen months. Model performance ranged from R^2^ = 0.24–0.32, with calibration slopes of 0.89–0.92 and intercepts of 31.30–449.45. Impaired QMVC (≤70% predicted) was also associated with fewer steps/day (β = −1251.39 [−2661.69; 158.91], R^2^ = 0.32, slope = 0.91, intercept = 449.45). Full model outputs are presented in Supplementary Materials, Tables S3, S9 and S12.

### 3.4. Secondary Outcomes

People with Long COVID consistently presented lower 6MWD, 1minSTS and QMVC than controls across the follow-up period (Table 2). Both groups showed significant improvements in the 6MWD (Long COVID: +19 [5; 33] m; controls: +30 [5; 56] m) and 1 min STS (Long COVID: +2 [1; 3] reps; controls: +3 [1; 5] reps) between twelve and eighteen months. The proportion of people with Long COVID with impaired 6MWD (27% to 17%) and 1 min STS (37% to 33%) significantly decreased. No significant changes in QMVC were observed in either group throughout the follow-up period (Table 2). A significantly higher proportion of participants with Long COVID experienced clinically relevant dyspnoea, fatigue, symptoms of anxiety and depression, and impaired HRQoL compared to controls throughout the follow-up period (Table 2). Although the proportion of people with Long COVID reporting fatigue significantly decreased from twelve to eighteen months (83% to 67%), a substantial proportion remained symptomatic (dyspnoea 24%, fatigue 67%, anxiety 47%, depression 26%), and 50% reported impaired HRQoL at the end of follow-up.

### 3.5. Exploratory Analyses

Hospitalised (HW, ICU) and non-hospitalised (H) participants’ baseline characteristics are presented in the Supplementary Materials. Hospitalised people were older, predominantly male, more comorbid and medicated, and had higher body mass index and worse lung function compared to those not hospitalised (Table S13).

Only hospitalised participants significantly reduced their time spent in moderate-to-vigorous PA (ICU −7 [−12; −2] min·day^−1^; HW −5 [−45; −2] min·day^−1^) between twelve and eighteen months. ICU-admitted participants significantly decreased their sedentary time from twelve to fifteen months (−68 [−133; −3] min·day^−1^). The proportion of participants walking fewer than 5000 steps·day^−1^ increased in all groups, being significant only in the non-hospitalised group (ICU 32% to 49%, HW 35% to 56%, H 34% to 66%). No other significant changes were observed in PA and sedentary behaviour measures (Table S14 and Figure S1).

Hospitalised participants consistently showed lower 6MWD and 1 min STS performance compared to non-hospitalised participants throughout the follow-up period (Table S14). All groups showed significant improvements in the 1minSTS (ICU +2 [1; 4] reps, HW +1 [0; 3] reps; H +2 [1; 4] reps), whereas only the non-hospitalised group showed a significant improvement in the 6MWD (ICU +15 [−10; 40] m, HW +11 [−13; 35] m, H +35 [10; 59] m). No other significant differences were detected across groups (Table S14).

By the end of follow-up, a substantial proportion of participants reported persistent dyspnoea (ICU 18%, HW 19%, H 11%), fatigue (ICU 52%, HW 53%, H 46%), symptoms of anxiety (ICU 15%, HW 44%, H 46%) and depression (ICU 12%, HW 28%, H 14%), and impaired HRQoL (ICU 36%, HW 50%, H 23%; Table S14).

## 4. Discussion

This study explored PA levels, sedentary behaviour and potential behaviour-change contributing factors in people with and without Long COVID, from twelve to eighteen months after discharge. All participants remained physically inactive, although people with Long COVID increased light PA, while decreasing moderate-to-vigorous PA and sedentary time, adjusting to fit lower PA levels. At eighteen months, almost one third presented impaired functional capacity, a quarter to over a half of them reported persistent symptoms, particularly fatigue, and up to half of them reported impaired. Dyspnoea and impaired peripheral muscle strength were key contributing factors to decreased light PA and step count, as well as increased sedentary time.

Our findings align with previous research reporting high physical inactivity rates (67%) among hospitalised patients after COVID-19 within twelve months from infection, regardless of initial severity [16]. Furthermore, a high physical inactivity proportions (70%) and low step counts have been reported among people with Long COVID (15 ± 10 months from acute disease), particularly when affected by fatigue, post-exertional malaise and co-occurrence of ≥2 physical symptoms (affected vs. not affected: 6707 ± 3570 vs. 12,050 ± 5636 steps·day^−1^, 5993 ± 2500 vs. 9271 ± 5630 steps·day^−1^, 6464 ± 2665 vs. 12,290 ± 5872 steps·day^−1^, respectively) [46].

In our cohort, people with Long COVID from twelve to eighteen months, consistently decreased daily time spent in moderate-to-vigorous PA (12 ± 1 to 7 ± 1 min·day^−1^) and step count (4684 ± 3578 to 3847 ± 2599 steps·day^−1^), with proportions of people walking fewer than 5000 steps·day^−1^ increasing (36% to 61%). At the same time, high proportions of people with Long COVID reported persistent symptoms and up to 50% impaired HRQoL. These results support a deconditioning cycle, where symptom avoidance reduces PA volume or intensity, and in turn, physical inactivity exacerbates symptoms, further limiting PA [13]. Indeed, symptom exacerbations follow cycling patterns, triggered by physical effort, and overlap with post-exertional malaise episodes, reducing PA and worsening physical condition and symptoms in people with Long COVID [47,48].

Persisting dyspnoea in Long COVID may stem from increased neural drive due to pathophysiological factors (e.g., increased mechanical load on respiratory muscles, heightened chemoreceptor activation in response to higher protons concentration—mitochondrial dysfunction, anaerobic metabolism in peripheral muscles [49], increased heart and respiratory rates—autonomic dysregulation) [50] and psychological burden (i.e., anticipatory fear and dyspnoea-related anxiety) [51]. These mechanisms may contribute to PA avoidance and worsening symptoms, as seen in other chronic diseases [52]. Recent research correlates dyspnoea with fatigue and reduced functional capacity, characterising a dyspnoea-related phenotype in Long COVID with pronounced fatigue, independent of lung function [53]. In line with these findings, in our study, the impaired functional capacity and peripheral muscle strength, persistent fatigue and anxiety may have further contributed to dyspnoea and PA reduction in people with Long COVID.

The sustained PA and sedentary behaviour change, and high symptom burden suggest that current therapeutic strategies may not fully meet individual needs. While (pulmonary) rehabilitation can improve symptoms and functional capacity in people with Long COVID [54,55], its impact on PA levels remains inconsistent [56]. Interventions should prioritise long-term deconditioning prevention through PA programmes, successful in other chronic disease populations [57,58], along with fatigue management strategies, such as pacing and gradual activity exposure [59]. Multidisciplinary approaches offering integrated physical and psychological support may enhance symptom management.

From a public health perspective, persistent inactivity and symptoms in people with Long COVID may result in a significant long-term burden. To address this, initiatives should promote safe activity pacing, integrate pulmonary rehabilitation within primary and community care, and improve access to comprehensive support services. Large-scale efforts such as the National COVID Cohort Collaborative-N3C and the Researching COVID to Enhance Recovery-RECOVER programme in the United States [60,61], as well as the Spanish Network for Research on Long COVID-REiCOP [62], exemplify strategies to characterise Long COVID and inform responsive health policy. As highlighted by recent global consensus, targeted investments and tailored policies are urgently needed to mitigate the Long COVID impact on society and the economy, and to support long-term recovery [63].

### Strengths and Limitations

To our knowledge, this is the first study to longitudinally and objectively assess PA and sedentary behaviour in people with Long COVID, twelve months after discharge, across different acute disease severity, from the first COVID-19 outbreak. Efforts were made to minimise bias by excluding pre-infection vaccinated individuals and those with reinfections. PA and sedentary time were objectively measured using validated inertial systems and a machine learning algorithm.

However, several limitations must be acknowledged. The absence of pre-infection data limited our ability to define PA changes accurately. The follow-up starting at twelve months may have overlooked early recovery or complications, although it helped to capture more stable trends. A six-month follow-up period may be relatively short for exploring long-term PA behaviours, given that seasonal and environmental factors influence, but adjustments for daylight exposure and diverse setting recruitment (rural; urban) were applied. The lack of a healthy control group limits generalizability, but many potential volunteers contracted COVID-19 during recruitment, making them unsuitable for comparison. Furthermore, repeated assessments over consecutive days could have provided more accurate insights into physical impairments and symptoms, as previously described [64]; however, logistic constraints (e.g., time-consuming data collection, participant travel and associated costs) made this unfeasible.

Future research should include healthy controls and pre-infection data to better quantify the Long COVID impact. Tailored interventions integrating physical and psychological components, including behaviour-change strategies, should be explored to effectively address persistent symptoms and PA limitations in people with Long COVID.

## 5. Conclusions

Although all people remain physically inactive even long after COVID-19, people with Long COVID adjust their PA intensity and volume to fit lower PA levels. While functional capacity generally improves over time, persistent symptoms may negatively impact HRQoL. Dyspnoea and impaired peripheral muscle strength may be associated with PA and sedentary behaviour changes. Tailored interventions, incorporating behaviour-change programmes, should be explored to prevent long-term deconditioning and address patient-specific needs.

## Figures and Tables

**Table 1:** Participants’ characteristics at twelve months after COVID-19.

	All	Control Group	Long COVID Group	Sig.
Participants n	148	47	101	
Demographic and anthropometric data				
Age (years) mean ± SD	58 ± 15	54 ± 17	60 ± 14	*p* = 0.04 *
Male	80 (54)	31 (66)	49 (49)	*p* = 0.05
BMI (kg·m^−2^) mean ± SE	27.9 ± 0.4	27.0 ± 0.7	28.8 ± 0.5	*p* = 0.30
Lung function				
FEV_1_%predicted mean ± SE	90 ± 1	92 ± 2	87 ± 2	*p* = 0.76
FVC%predicted mean ± SE	86 ± 1	88 ± 2	84 ± 2	*p* = 0.65
Medical records				
Comorbidities median (Q1; Q3)	2 (1; 3)	1 (0; 2)	2 (1; 3)	*p* < 0.01 *
Endocrine or metabolic diseases ^¶^ n (%)	60 (41)	11 (23)	49 (49)	*p* < 0.01 *
Circularity system diseases ^¶^ n (%)	59 (40)	18 (38)	41 (41)	*p* = 0.86
Musculoskeletal system diseases ^¶^ n (%)	30 (20)	6 (13)	24 (24)	*p* = 0.13
Respiratory system diseases ^¶^ n (%)	22 (15)	4 (9)	18 (18)	*p* = 0.21
Immune system diseases ^¶^ n (%)	17 (12)	4 (9)	13 (13)	*p* = 0.58
Nervous system diseases ^¶^ n (%)	12 (8)	2 (4)	10 (10)	*p* = 0.34
Others ^¶^ n (%)	33 (22)	9 (19)	24 (24)	*p* = 0.67
Charlson comorbidity index median (Q1; Q3)	2 (0; 3)	2 (0; 3)	2 (1; 3)	*p* = 0.09
Charlson comorbidity categories (mild/moderate/severe)	53 (36)/48 (32)/8 (5)	16 (34)/15 (32)/0 (0)	37 (37)/33 (33)/8 (8)	*p* = 0.14
Medications median (Q1; Q3)	1 (0; 2)	1 (0; 1)	1 (1; 2)	*p* < 0.01 *
Cardiovascular system ^#^ n (%)	65 (44)	19 (40)	46 (46)	*p* = 0.60
Alimentary track and metabolism ^#^ n (%)	25 (17)	3 (6)	22 (22)	*p* = 0.02 *
Respiratory system ^#^ n (%)	20 (14)	1 (2)	14 (14)	*p* = 0.30
Systemic hormonal preparations, and insulins ^#^ n (%)	19 (13)	1 (2)	18 (18)	*p* < 0.01 *
Nervous system ^#^ n (%)	15 (10)	1 (2)	3 (3)	*p* = 0.04 *
Others ^#^	13 (9)	3 (6)	10 (10)	*p* = 0.76

All data, reported as mean ± SE, are adjusted for age and sex. Abbreviations: BMI: body mass index; COVID: Coronavirus Disease 2019; FEV1: forced expiratory volume in one second; FVC: forced vital capacity. * Statistically significant *p*-value < 0.05 between groups. ^¶^ International Classification of Diseases, 11th Ed. Others include infectious diseases, ear disorders, digestive system disorders, genitourinary system diseases, developmental anomalies, sleep-awake disorders, mental disorders and symptoms and signs not elsewhere classified. ^#^ Anatomical Therapeutic Chemical Classification System. Others include the dermatological, musculoskeletal system, and blood and blood-forming organs.

**Table 2:** Primary and secondary outcomes in people with and without Long COVID at twelve, fifteen and eighteen months after discharge.

	All ^#^			Control Group ^#^			Long COVID Group ^#^			
	Twelve Months	Fifteen Months	Eighteen Months	Twelve Months	Fifteen Months	Eighteen Months	Twelve Months	Fifteen Months	Eighteen Months	Sig.
Participants n	148	117	104	47	36	32	101	81	72	
Primary outcomes										
LPA (min·day^−1^)	272 ± 13	260 ± 12	277 ± 12	290 ± 22	268 ± 20	275 ± 20	253 ± 16	251 ± 13	279 ± 14 ^†^	*p* = 0.29
MVPA (min·day^−1^)	11 ± 1	8 ± 1 ^†^	7 ± 1 ^†^	11 ± 2	8 ± 1	7 ± 1	12 ± 1	8 ± 1 ^†^	7 ± 1 ^†^	*p* = 0.46
Steps·day^−1^	5306 ± 301	4541 ± 252 ^†^	4513 ± 270 ^†^	5927 ± 486	5062 ± 412	5179 ± 446	4684 ± 356	4021 ± 285	3847 ± 306	*p* = 0.81
Steps·day^−1^ ≤ 5000 n (%)	50 (34)	62 (53)	59 (57) ^†^	14 (30)	18 (50)	15 (47)	36 (36)	44 (54)	44 (61) ^†^	*p* = 0.61
Sedentary time (min·day^−1^)	425 ± 15	387 ± 12 ^†^	378 ± 14 ^†^	416 ± 24	386 ± 19	366 ± 22	434 ± 18	387 ± 13 ^†^	390 ± 15	*p* = 0.61
Secondary outcomes										
6MWD (m)	578 ± 12	597 ± 12 ^†^	601 ± 13 ^†^	632 ± 19	653 ± 20 ^†^	663 ± 22 ^†^	523 ± 13	542 ± 13 ^†^	538 ± 15	*p* = 0.44
6MWD ≤ 70% predicted n (%)	30 (20)	19 (16)	12 (11)	3 (6)	1 (3)	0 (0)	27 (27)	18 (22) ^†^	12(17) ^†^	*p* = 0.48
1 min STS (reps)	26 ± 1	28 ± 1 ^†^	29 ± 1 ^†^	30 ± 1	31 ± 1 ^†^	33 ± 2 ^†^	23 ± 1	25 ± 1 ^†^	25 ± 1 ^†^	*p* = 0.11
1minSTS ≤ 70% predicted n (%)	44 (30)	29 (25)	28 (27)	7 (15)	4 (11)	4 (13)	37 (37)	25 (31)	24 (33) ^†^	*p* = 0.11
QMVC (kgf)	18 ± 1	18 ± 1	18 ± 1	20 ± 1	20 ± 1	21 ± 1	15 ± 1	15 ± 1	16 ± 1	*p* = 0.37
QMVC ≤ 70% predicted n (%)	26 (18)	19 (16)	20 (19)	2 (4)	1 (3)	2 (6)	24 (24)	18 (22)	18 (25)	*p* = 0.95
Dyspnoea mMRC	1 (0; 1)	1 (0; 1)	1 (0; 1)	0 (0; 1)	0 (0; 1)	0 (0; 1)	1 (0; 1)	1 (1; 1)	1 (0; 1)	*p* = 0.94
mMRC ≥ 2 n (%)	18 (12)	14 (12)	17 (16)	1 (2)	0 (0) ^†^	0 (0) ^†^	18 (18)	14 (17)	17 (24)	*p* = 0.42
Fatigue FACIT-FS	42 (33; 49)	43 (34; 48)	44 (35; 50) ^†^	50 (48; 51)	50 (47; 51)	50 (48; 52)	37 (30; 42)	38 (30; 45)	38 (31; 46)	*p* = 0.24
FACIT-FS ≤ 43 n (%)	84 (57)	60 (51) ^†^	52 (50) ^†^	0 (0)	4 (11)	4 (13)	84 (83)	56 (69) ^†^	48 (67) ^†^	*p* < 0.01 *
Anxiety HADS-A	4 (2; 7)	5 (2; 7)	4 (2; 8)	2 (1; 4)	2 (0; 4)	2 (1; 4)	6 (3; 9)	6 (3; 8)	6 (3; 9)	*p* = 0.84
HADS-A ≥ 8 n (%)	41 (28)	37 (32) ^†^	37 (36) ^†^	0 (0)	3 (8)	3 (9)	41 (41)	34 (42)	34 (47)	*p* < 0.01 *
Depression HADS-D	4 (3; 6)	3 (1; 6) ^†^	3 (1; 6) ^†^	3 (1; 4)	1 (0; 2) ^†^	1 (0; 2) ^†^	5 (3; 7)	4 (3; 7)	4 (3; 7)	*p* = 0.09
HADS-D ≥ 8 n (%)	29 (20)	23 (20)	19 (18)	0 (0)	1 (3)	0 (0)	29 (29)	22 (27)	19 (26)	*p* < 0.01 *
EQ-5D-5L	80 ± 1	79 ± 1	80 ± 2	90 ± 2	86 ± 2	88 ± 3	70 ± 2	72 ± 2	73 ± 2	*p* = 0.22
EQ-5D-5L ≤ 74/female or 78/male n (%)	60 (41)	45 (38)	38 (37) ^†^	0 (0)	2 (5)	2 (6)	60 (59)	43 (53)	36 (50)	*p* < 0.01 *

Data expressed as mean ± SE or median (Q1; Q3), unless otherwise reported. All data are adjusted for age and sex. Abbreviations. 1minSTS: 1-min sit-to-stand test; 6MWD: 6-min walking distance; EQ-5D-5L: European quality of life—5 dimensions—5 levels; FACIT-FS: functional assessment of chronic illness therapy—fatigue; HADS: hospital anxiety and depression scale; LPA: time spent in light physical activity; mMRC: modified Medical Research Council; MVPA: time spent in moderate-to-vigorous physical activity; QMVC: quadriceps muscle voluntary contraction. * Statistically significant *p*-value < 0.017 between groups, as follows. At twelve months: Steps·day^−1^: *p* = 0.041; 6MWD: *p* < 0.001; 6MWD ≤ 70% predicted: *p* = 0.001; 1 min STS: *p* < 0.001; 1 min STS ≤ 70% predicted: *p* = 0.012; QMVC: *p* < 0.001; QMVC ≤ 70% predicted: *p* = 0.015; mMRC ≥ 2: *p* = 0.002; FACIT-FS ≤ 43: *p* < 0.001; HADS-A ≥ 8: *p* < 0.001; HADS-D ≥ 8: *p* < 0.001; EQ-5D-5L: *p* < 0.001; EQ-5D-5L ≤ 74/female or 78/male: *p* < 0.001. At fifteen months: Steps·day^−1^: *p* = 0.041; 6MWD: *p* < 0.001; 6MWD ≤ 70% predicted: *p* = 0.001; 1 min STS: *p* < 0.001; 1 min STS ≤ 70% predicted: *p* = 0.023; QMVC: *p* < 0.001; QMVC ≤ 70% predicted: *p* = 0.012; mMRC ≥ 2: *p* < 0.001; FACIT-FS ≤ 43: *p* < 0.001; HADS-A ≥ 8: *p* < 0.001; HADS-D ≥ 8: *p* = 0.002; EQ-5D-5L: *p* < 0.001; EQ-5D-5L ≤ 74/female or 78/male: *p* < 0.001. At eighteen months: Steps·day^−1^: *p* = 0.016; 6MWD: *p* < 0.001; 6MWD ≤ 70% predicted: *p* = 0.004; 1min STS: *p* < 0.001; QMVC: *p* < 0.001; QMVC ≤ 70% predicted: *p* = 0.014; mMRC ≥ 2: *p* = 0.001; FACIT-FS ≤ 43: *p* < 0.001; HADS-A ≥ 8: *p* < 0.001; HADS-D ≥ 8: *p* < 0.001; EQ-5D-5L: *p* < 0.001; EQ-5D-5L ≤ 74/female or 78/male: *p* < 0.001. † Statistically significant *p*-value < 0.05 within each group, as follows. In All: MVPA: *p* = 0.005 between twelve and fifteen months, *p* < 0.001 between twelve and eighteen months; Steps·day^−1^: *p* = 0.028 between twelve and fifteen months, *p* = 0.046 between twelve and eighteen months; Steps·day^−1^ ≤ 5000: *p* < 0.034 between twelve and eighteen months; Sedentary time: *p* = 0.032 between twelve and fifteen months, *p* = 0.013 between twelve and eighteen months; 6MWD: *p* < 0.001 between twelve and fifteen months, *p* = 0.001 between twelve and eighteen months; 1minSTS: *p* = 0.004 between twelve and fifteen months, *p* < 0.001 between twelve and eighteen months, *p* = 0.009 between fifteen and eighteen months; FACIT-FS: *p* = 0.001 between twelve and fifteen months, *p* < 0.001 between twelve and eighteen months; HADS-A ≥ 8: *p* = 0.001 between twelve and fifteen months, *p* = 0.011 between twelve and eighteen months; HADS-D: *p* = 0.025 between twelve and fifteen months, *p* = 0.031 between twelve and eighteen months; EQ-5D-5L ≤ 74/female or 78/male: *p* = 0.012 between twelve and eighteen months; In Control group: 6MWD: *p* = 0.046 between twelve and fifteen months, *p* = 0.014 between twelve and eighteen months; 1 min STS: *p* < 0.001 between twelve and fifteen months, *p* = 0.009 between fifteen and eighteen months; mMRC ≥ 2: *p* < 0.001 between twelve and fifteen months, twelve and eighteen months, and fifteen and eighteen months. In Long COVID group: LPA: *p* = 0.038 between fifteen and eighteen months; MVPA: *p* = 0.022 between twelve and fifteen months, *p* = 0.001 between twelve and eighteen months; Steps·day^−1^ ≤ 5000: *p* = 0.034 between twelve and eighteen months; Sedentary time: *p* = 0.027 between twelve and fifteen months; 6MWD: *p* = 0.003 between twelve and fifteen months; 6MWD ≤ 70% predicted: *p* < 0.001 between twelve and fifteen months, *p* = 0.015 between twelve and eighteen months, *p* = 0.015 between fifteen and eighteen months; 1 min STS ≤ 70% predicted: *p* = 0.027 between twelve and eighteen months; FACIT-FS ≤ 43: *p* = 0.001 between twelve and fifteen months, *p* = 0.001 between twelve and eighteen months. # Missing values, as follows. In Control group: At twelve months: LPA (n = 11), MVPA (n = 12), Steps·day^−1^ (n = 12), Sedentary time (n = 12); At fifteen months: LPA (n = 4), MVPA (n = 5), Steps·day^−1^ (n = 5), Sedentary time (n = 4); At eighteen months: LPA (n = 5), MVPA (n = 5), Steps·day^−1^ (n = 5), Sedentary time (n = 6); In Long COVID group: At twelve months: LPA (n = 35), MVPA (n = 37), Steps·day^−1^ (n = 38), Sedentary time (n = 37); At fifteen months: LPA (n = 15), MVPA (n = 15), Steps·day^−1^ (n = 15), Sedentary time (n = 15); At eighteen months: LPA (n = 12), MVPA (n = 12), Steps·day^−1^ (n = 15), Sedentary time (n = 13).

## Data Availability

Data available upon reasonable request to the corresponding author.

## Supplementary Materials

The following supporting information can be downloaded at: https://www.mdpi.com/article/10.3390/jcm14113641/s1, Table S1: People after COVID-19 at twelve months post-discharge, grouped by time spent in light physical activity change over 6-month follow-up; Table S2: Correlations between time spent in light physical activity at eighteen months and all baseline potential predictors; Table S3: Potential predictors for time spent in light physical activity eighteen months, starting at twelve months post-COVID-19—Stepwise regression method vs. LASSO regression method; Table S4: People after COVID-19 at twelve months post-discharge, grouped by time spent in moderate-to-vigorous change over 6-month follow-up; Table S5: Correlations between time spent in moderate-to-vigorous physical activity at eighteen months and all baseline potential predictors; Table S6: Potential predictors for time spent in moderate-to-vigorous physical activity at eighteen months, starting at twelve months post-COVID-19—Stepwise regression method vs. LASSO regression method; Table S7: People after COVID-19 at twelve months post-discharge, grouped by steps per day change over 6-month follow-up; Table S8: Correlations between steps per day at eighteen months and all baseline potential predictors; Table S9: Potential predictors for steps per day at eighteen months, starting at twelve months post-COVID-19—Stepwise regression method vs. LASSO regression method; Table S10: People after COVID-19 at twelve months post-discharge, grouped by sedentary time change over 6-month follow-up; Table S11: Correlations between sedentary time at eighteen months and all baseline potential predictors; Table S12: Potential predictors for sedentary time at eighteen months, starting at twelve months post-COVID-19—Stepwise regression method vs. LASSO regression method; Table S13:. Participants’ characteristics at twelve months after COVID-19, exploratory analyses; Table S14: Primary and secondary outcomes in people after COVID-19, at twelve, fifteen and eighteen months after discharge, exploratory analyses; Figure S1: Physical activity and sedentary behaviour changes of people after COVID-19, during six-month follow-up, starting from twelve months post-discharge, exploratory analyses.

## Abbreviations

The following abbreviations are used in this manuscript:

1minSTS	1-min sit-to-stand test
6MWD	6-min walk test
COVID-19	Coronavirus Disease 2019
EQ-5D-5L	European quality of life—5 Dimensions—5 Levels
FACIT-FS	Functional Assessment of Chronic Illness Therapy—Fatigue
HADS	Hospital Anxiety and Depression Scale
HRQoL	Health-related quality of life
mMRC	Modified Medical Research Council
PA	Physical activity
QMVC	Quadriceps muscle voluntary contraction

## References

[1] The Lancet Respiratory Medicine (2023). Long COVID: Confronting a Growing Public Health Crisis. Lancet Respir. Med..

[2] Soriano J.B., Murthy S., Marshall J.C., Relan P., Diaz J.V. (2022). A Clinical Case Definition of Post-COVID-19 Condition by a Delphi Consensus. Lancet Infect. Dis..

[3] Hou Y., Gu T., Ni Z., Shi X., Ranney M.L., Mukherjee B. (2025). Global Prevalence of Long COVID, Its Subtypes and Risk Factors: An Updated Systematic Review and Meta-Analysis. medRxiv.

[4] Sudre C.H., Murray B., Varsavsky T., Graham M.S., Penfold R.S., Bowyer R.C., Pujol J.C., Klaser K., Antonelli M., Canas L.S. (2021). Attributes and Predictors of Long COVID. Nat. Med..

[5] Singh S.J., Baldwin M.M., Daynes E., Evans R.A., Greening N.J., Jenkins R.G., Lone N.I., McAuley H., Mehta P., Newman J. (2023). Respiratory Sequelae of COVID-19: Pulmonary and Extrapulmonary Origins, and Approaches to Clinical Care and Rehabilitation. Lancet Respir. Med..

[6] Castanares-Zapatero D., Chalon P., Kohn L., Dauvrin M., Detollenaere J., Maertens de Noordhout C., Primus-de Jong C., Cleemput I., Van den Heede K. (2022). Pathophysiology and Mechanism of Long COVID: A Comprehensive Review. Ann. Med..

[7] Shah D.P., Thaweethai T., Karlson E.W., Bonilla H., Horne B.D., Mullington J.M., Wisnivesky J.P., Hornig M., Shinnick D.J., Klein J.D. (2025). Sex Differences in Long COVID. JAMA Netw. Open.

[8] Mansell V., Hall Dykgraaf S., Kidd M., Goodyear-Smith F. (2022). Long COVID and Older People. Lancet Healthy Longev..

[9] Lopez-Leon S., Wegman-Ostrosky T., Perelman C., Sepulveda R., Rebolledo P.A., Cuapio A., Villapol S. (2021). More Than 50 Long-Term Effects of COVID-19: A Systematic Review and Meta-Analysis. Sci. Rep..

[10] Fernandez-de-las-Peñas C., Palacios-Cena D., Gomez-Mayordomo V., Florencio L.L., Cuadrado M.L., Plaza-Manzano G., Navarro-Santana M. (2021). Prevalence of Post-COVID-19 Symptoms in Hospitalized and Non-Hospitalized COVID-19 Survivors: A Systematic Review and Meta-Analysis. Eur. J. Intern. Med..

[11] Bahmer T., Borzikowsky C., Lieb W., Horn A., Krist L., Fricke J., Scheibenbogen C., Rabe K.F., Maetzler W., Maetzler C. (2022). Severity, Predictors and Clinical Correlates of Post-COVID Syndrome (PCS) in Germany: A Prospective, Multi-Centre, Population-Based Cohort Study. EClinicalMedicine.

[12] Torres M., Serra-Sutton V., Soriano J.B., Ferrer M., Trejo A., Benavides F.G., Lumbreras B., Pérez-Gómez B., Pijoan J.I., Monguet J.M. (2023). Consensus on Post COVID in the Spanish National Health System: Results of the CIBERPOSTCOVID EDelphi Study. J. Infect. Public Health.

[13] Lippi G., Mattiuzzi C., Sanchis-Gomar F. (2024). Physical Activity, Long-COVID, and Inactivity: A Detrimental Endless Loop. J. Phys. Act. Health.

[14] Tanriverdi A., Savci S., Kahraman B.O., Ozpelit E. (2022). Extrapulmonary Features of Post-COVID-19 Patients: Muscle Function, Physical Activity, Mood, and Sleep Quality. Ir. J. Med. Sci..

[15] Delbressine J.M., Machado F.V.C., Goërtz Y.M.J., Van Herck M., Meys R., Houben-Wilke S., Burtin C., Franssen F.M.E., Spies Y., Vijlbrief H. (2021). The Impact of Post-COVID-19 Syndrome on Self-Reported Physical Activity. Int. J. Environ. Res. Public Health.

[16] Plekhanova T., Rowlands A.V., Evans R.A., Edwardson C.L., Bishop N.C., Bolton C.E., Chalmers J.D., Davies M.J., Daynes E., Dempsey P.C. (2022). Device-Assessed Sleep and Physical Activity in Individuals Recovering from a Hospital Admission for COVID-19: A Multicentre Study. Int. J. Behav. Nutr. Phys. Act..

[17] Ballouz T., Menges D., Anagnostopoulos A., Domenghino A., Aschmann H.E., Frei A., Fehr J.S., Puhan M.A. (2023). Recovery and Symptom Trajectories up to Two Years After SARS-CoV-2 Infection: Population Based, Longitudinal Cohort Study. BMJ.

[18] Lee I.-M., Shiroma E.J., Lobelo F., Puska P., Blair S.N., Katzmarzyk P.T. (2012). Impact of Physical Inactivity on the World’s Major Non-Communicable Diseases. Lancet.

[19] Vandenbroucke J.P., Von Elm E., Altman D.G., Gøtzsche P.C., Mulrow C.D., Pocock S.J., Poole C., Schlesselman J.J., Egger M. (2007). Strengthening the Reporting of Observational Studies in Epidemiology (STROBE): Explanation and Elaboration. Epidemiology.

[20] Collins G.S., Reitsma J.B., Altman D.G., Moons K.G.M. (2015). Transparent Reporting of a Multivariable Prediction Model for Individual Prognosis or Diagnosis (TRIPOD): The TRIPOD Statement. BMJ.

[21] Demeyer H., Mohan D., Burtin C., Vaes A.W., Heasley M., Bowler R.P., Casaburi R., Cooper C.B., Corriol-Rohou S., Frei A. (2021). Objectively Measured Physical Activity in Patients with COPD: Recommendations from an International Task Force on Physical Activity. Chronic Obstr. Pulm. Dis..

[22] Yarowsky D. Unsupervised Word Sense Disambiguation Rivaling Supervised Methods. Proceedings of the Annual Meeting of the Association for Computational Linguistics.

[23] Schölkopf B., Smola A.J. (2018). Learning with Kernels: Support Vector Machines, Regularization, Optimization, and Beyond.

[24] Muhsen H., Al-Amaydeh O., Al-Hamlan R. (2020). Algorithm Design for Accurate Steps Counting Based on Smartphone Sensors for Indoor Applications. Adv. Sci. Technol. Eng. Syst..

[25] Ainsworth B.E., Haskell W.L., Herrmann S.D., Meckes N., Bassett D.R., Tudor-Locke C., Greer J.L., Vezina J., Whitt-Glover M.C., Leon A.S. (2011). 2011 Compendium of Physical Activities: A Second Update of Codes and MET Values. Med. Sci. Sports Exerc..

[26] Lluva-Plaza S., Jiménez-Martín A., Gualda-Gómez D., Villadangos-Carrizo J.M., García-Domínguez J.J. (2023). Multisensory System for Long-Term Activity Monitoring to Facilitate Aging-in-Place. Sensors.

[27] Holland A.E., Spruit M.A., Troosters T., Puhan M.A., Pepin V., Saey D., McCormack M.C., Carlin B.W., Sciurba F.C., Pitta F. (2014). An Official European Respiratory Society/American Thoracic Society Technical Standard: Field Walking Tests in Chronic Respiratory Disease. Eur. Respir. J..

[28] Crook S., Büsching G., Schultz K., Lehbert N., Jelusic D., Keusch S., Wittmann M., Schuler M., Radtke T., Frey M. (2017). A Multicentre Validation of the 1-Min Sit-to-Stand Test in Patients with COPD. Eur. Respir. J..

[29] Lesnak J., Anderson D., Farmer B., Katsavelis D., Grindstaff T.L. (2019). Validity of Hand-Held Dynamometry in Measuring Quadriceps Strength and Rate of Torque Development. Int. J. Sports Phys. Ther..

[30] Bohannon R.W., Kindig J., Sabo G., Duni A.E., Cram P. (2012). Isometric Knee Extension Force Measured Using a Handheld Dynamometer with and Without Belt-Stabilization. Physiother. Theory Pract..

[31] Gimeno-Santos E., Arbillaga-Etxarri A., Vilaró J., Balañá A., Barberán-Garcia A., Del Corral-Núñez T., Fernández J.C., Jiménez B., López A., López D. (2015). Reference Equations for 6-Minute Walk Test in Spanish Population. Eur. Respir. J..

[32] Vilarinho R., Montes A.M., Noites A., Silva F., Melo C. (2024). Reference Values for the 1-Minute Sit-to-Stand and 5 Times Sit-to-Stand Tests to Assess Functional Capacity: A Cross-Sectional Study. Physiotherapy.

[33] Tanguay S., Saey D., Marklund S., Nyberg A., Gephine S., Frykholm E., De Brandt J., Burtin C., Maltais F. (2023). Reference Equations for Quadriceps Strength, Endurance and Power: A Multicentre Study. ERJ Open Res..

[34] Bestall J.C., Paul E.A., Garrod R., Garnham R., Jones P.W., Wedzicha J.A. (1999). Usefulness of the Medical Research Council (MRC) Dyspnoea Scale as a Measure of Disability in Patients with Chronic Obstructive Pulmonary Disease. Thorax.

[35] Hewlett S., Dures E., Almeida C. (2011). Measures of Fatigue: Bristol Rheumatoid Arthritis Fatigue Multi-Dimensional Questionnaire (BRAF MDQ), Bristol Rheumatoid Arthritis Fatigue Numerical Rating Scales (BRAF NRS) for Severity, Effect, and Coping, Chalder Fatigue Questionnaire (CFQ), Checklist. Arthritis Care Res..

[36] Bjelland I., Dahl A.A., Haug T.T., Neckelmann D. (2002). The Validity of the Hospital Anxiety and Depression Scale. J. Psychosom. Res..

[37] Hernandez G., Garin O., Pardo Y., Vilagut G., Pont À., Suárez M., Neira M., Rajmil L., Gorostiza I., Ramallo-Fariña Y. (2018). Validity of the EQ–5D–5L and Reference Norms for the Spanish Population. Qual. Life Res..

[38] Garcia-Gordillo M.A., Adsuar J.C., Olivares P.R. (2016). Normative Values of EQ-5D-5L: In a Spanish Representative Population Sample from Spanish Health Survey, 2011. Qual. Life Res..

[39] Harrison J.E., Weber S., Jakob R., Chute C.G. (2021). ICD-11: An International Classification of Diseases for the Twenty-First Century. BMC Med. Inform. Decis. Mak..

[40] Charlson M.E., Pompei P., Ales K.L., MacKenzie C.R. (1987). A New Method of Classifying Prognostic Comorbidity in Longitudinal Studies: Development and Validation. J. Chronic Dis..

[41] WHO (2023). Collaborating Centre for Drug Statistics Methodology, Guidelines for ATC Classification and DDD Assignment 2024.

[42] Graham B.L., Steenbruggen I., Barjaktarevic I.Z., Cooper B.G., Hall G.L., Hallstrand T.S., Kaminsky D.A., McCarthy K., McCormack M.C., Miller M.R. (2019). Standardization of Spirometry 2019 Update an Official American Thoracic Society and European Respiratory Society Technical Statement. Am. J. Respir. Crit. Care Med..

[43] Collins G.S., Dhiman P., Ma J., Schlussel M.M., Archer L., Van Calster B., Harrell F.E., Martin G.P., Moons K.G.M., van Smeden M. (2024). Evaluation of Clinical Prediction Models (Part 1): From Development to External Validation. BMJ.

[44] Bull F.C., Al-Ansari S.S., Biddle S., Borodulin K., Buman M.P., Cardon G., Carty C., Chaput J.P., Chastin S., Chou R. (2020). World Health Organization 2020 Guidelines on Physical Activity and Sedentary Behaviour. Br. J. Sports Med..

[45] Demeyer H., Burtin C., Hornikx M., Camillo C.A., Van Remoortel H., Langer D., Janssens W., Troosters T. (2016). The Minimal Important Difference in Physical Activity in Patients with COPD. PLoS ONE.

[46] Rosa-Souza F.J., Freire Y.A., Galliano L.M., Dalton-Alves F., de Lima Pinto J.C.B., Godtsfriedt C.E.S., Delevatti R.S., Gerage A.M., Rech C.R., Ritti-Dias R.M. (2024). Association of Physical Symptoms with Accelerometer-Measured Movement Behaviors and Functional Capacity in Individuals with Long COVID. Sci. Rep..

[47] Davis H.E., Assaf G.S., McCorkell L., Wei H., Low R.J., Re’em Y., Redfield S., Austin J.P., Akrami A. (2021). Characterizing Long COVID in an International Cohort: 7 Months of Symptoms and Their Impact. EClinicalMedicine.

[48] Appelman B., Charlton B.T., Goulding R.P., Kerkhoff T.J., Breedveld E.A., Noort W., Offringa C., Bloemers F.W., van Weeghel M., Schomakers B.V. (2024). Muscle Abnormalities Worsen after Post-Exertional Malaise in Long COVID. Nat. Commun..

[49] Davis H.E., McCorkell L., Vogel J.M., Topol E.J. (2023). Long COVID: Major Findings, Mechanisms and Recommendations. Nat. Rev. Microbiol..

[50] Freire A.P.C.F., Lira F.S., Morano A.E.v.A., Pereira T., Coelho-E-Silva M.-J., Caseiro A., Christofaro D.G.D., Júnior O.M., Dorneles G.P., Minuzzi L.G. (2022). Role of Body Mass and Physical Activity in Autonomic Function Modulation on Post-COVID-19 Condition: An Observational Subanalysis of Fit-COVID Study. Int. J. Environ. Res. Public Health.

[51] Daines L., Zheng B., Elneima O., Harrison E., Lone N.I., Hurst J.R., Brown J.S., Sapey E., Chalmers J.D., Quint J.K. (2023). Characteristics and Risk Factors for Post-COVID-19 Breathlessness after Hospitalisation for COVID-19. ERJ Open Res..

[52] Hanania N.A., O’Donnell D.E. (2019). Activity-Related Dyspnea in Chronic Obstructive Pulmonary Disease: Physical and Psychological Consequences, Unmet Needs, and Future Directions. Int. J. Chron. Obs. Pulmon Dis..

[53] Smith M.P., Sharpe H., Damant R.W., Ferrara G., Lim R.K., Stickland M.K., Lam G.Y. (2024). Factors Associated with Phenotypes of Dyspnea in Post-COVID-19 Condition: A Cross-Sectional Study. Sci. Rep..

[54] Oliveira M.R., Hoffman M., Jones A.W., Holland A.E., Borghi-Silva A. (2024). Effect of Pulmonary Rehabilitation on Exercise Capacity, Dyspnea, Fatigue, and Peripheral Muscle Strength in Patients with Post-COVID-19 Syndrome: A Systematic Review and Meta-Analysis. Arch. Phys. Med. Rehabil..

[55] Ortiz-Ortigosa L., Gálvez-Álvarez P., Viñolo-Gil M.J., Rodriguez-Huguet M., Góngora-Rodríguez J., Martín-Valero R. (2024). Effectiveness of Pulmonary Rehabilitation Programmes and/or Respiratory Muscle Training in Patients with Post-COVID Conditions: A Systematic Review. Respir. Res..

[56] Poppele I., Ottiger M., Stegbauer M., Schlesinger T., Müller K. (2024). Device-Assessed Physical Activity and Sleep Quality of Post-COVID Patients Undergoing a Rehabilitation Program. BMC Sports Sci. Med. Rehabil..

[57] Reilly C., Sails J., Stavropoulos-Kalinoglou A., Birch R.J., McKenna J., Clifton I.J., Peckham D., Birch K.M., Price O.J. (2023). Physical Activity Promotion Interventions in Chronic Airways Disease: A Systematic Review and Meta-Analysis. Eur. Respir. Rev..

[58] Megaritis D., Hume E., Chynkiamis N., Buckley C., Polhemus A.M., Watz H., Troosters T., Vogiatzis I. (2023). Effects of Pharmacological and Non-Pharmacological Interventions on Physical Activity Outcomes in COPD: A Systematic Review and Meta-Analysis. ERJ Open Res..

[59] Sanal-Hayes N.E.M., Mclaughlin M., Hayes L.D., Mair J.L., Ormerod J., Carless D., Hilliard N., Meach R., Ingram J., Sculthorpe N.F. (2023). A Scoping Review of ‘Pacing’ for Management of Myalgic Encephalomyelitis/Chronic Fatigue Syndrome (ME/CFS): Lessons Learned for the Long COVID Pandemic. J. Transl. Med..

[60] N3C—Home. https://covid.cd2h.org/.

[61] Home | RECOVER COVID Initiative. https://recovercovid.org/.

[62] REICOP | Red Española de Investigación en COVID Persistente. https://reicop.org/.

[63] Ewing A.G., Joffe D., Blitshteyn S., Brooks A.E.S., Wist J., Bar-Yam Y., Bilodeau S., Curtin J., Duncan R., Faghy M. (2025). Long COVID Clinical Evaluation, Research and Impact on Society: A Global Expert Consensus. Ann. Clin. Microbiol. Antimicrob..

[64] Keller B., Receno C.N., Franconi C.J., Harenberg S., Stevens J., Mao X., Stevens S.R., Moore G., Levine S., Chia J. (2024). Cardiopulmonary and Metabolic Responses During a 2-Day CPET in Myalgic Encephalomyelitis/Chronic Fatigue Syndrome: Translating Reduced Oxygen Consumption to Impairment Status to Treatment Considerations. J. Transl. Med..

