# Supplementary material for: Physical Activity and Sedentary Behaviour in People with Long COVID: A Follow-Up from 12 to 18 Months After Discharge
Source: J Clin Med. 2025 May 22;14(11):3641. doi: 10.3390/jcm14113641 (PMC12155931; doi:10.3390/jcm14113641)
Supplement: Supplementary file 1 [file jcm-14-03641-s001.zip › jcm-3653291-supplementary.docx]

**Physical Activity and Sedentary Behaviour in People with Long COVID: A Follow-Up from 12 to 18 Months after Discharge**

**Supplementary Materials**

**Index**

[**Results -Tables** 2](#_Toc198718429)

[**Table S1. People after COVID-19 at twelve-months post-discharge, grouped by time spent in light physical activity change over 6-month follow-up** 2](#_Toc198718430)

[**Table S2. Correlations between time spent in light physical activity at eighteen-months and all baseline potential predictors** 3](#_Toc198718431)

[**Table S3. Potential predictors for time spent in light physical activity eighteen-months, starting at twelve-months post-COVID-19 - Stepwise regression method vs. LASSO regression method** 4](#_Toc198718432)

[**Table S4. People after COVID-19 at twelve-months post-discharge, grouped by time spent in moderate-to-vigorous change over 6-month follow-up** 5](#_Toc198718433)

[**Table S5. Correlations between time spent in moderate-to-vigorous physical activity at eighteen-months and all baseline potential predictors** 6](#_Toc198718434)

[**Table S6. Potential predictors for time spent in moderate-to-vigorous physical activity at eighteen-months, starting at twelve-months post-COVID-19 - Stepwise regression method vs. LASSO regression method** 7](#_Toc198718435)

[**Table S7. People after COVID-19 at twelve-months post-discharge, grouped by steps per day change over 6-month follow-up** 8](#_Toc198718436)

[**Table S8. Correlations between steps per day at eighteen-months and all baseline potential predictors** 9](#_Toc198718437)

[**Table S9. Potential predictors for steps per day at eighteen-months, starting at twelve-months post-COVID-19 - Stepwise regression method vs. LASSO regression method** 10](#_Toc198718438)

[**Table S10. People after COVID-19 at twelve-months post-discharge, grouped by sedentary time change over 6-month follow-up** 11](#_Toc198718439)

[**Table S11. Correlations between sedentary time at eighteen-months and all baseline potential predictors** 12](#_Toc198718440)

[**Table S12. Potential predictors for sedentary time at eighteen-months, starting at twelve-months post-COVID-19 - Stepwise regression method vs. LASSO regression method** 13](#_Toc198718441)

[**Table S13. Participants’ characteristics at twelve-months after COVID-19, exploratory analyses** 14](#_Toc198718442)

[**Table S14. Primary and secondary outcomes in people after COVID-19, at twelve, fifteen and eighteen months after discharge, exploratory analyses** 15](#_Toc198718443)

[**Results – Figures** 17](#_Toc198718444)

[**Figure S1. Physical activity and sedentary behaviour changes of people after COVID-19, during six-month follow-up, starting from twelve-months post-discharge, exploratory analyses** 17](#_Toc198718445)

## **Results -Tables**

### **Table S1. People after COVID-19 at twelve-months post-discharge, grouped by time spent in light physical activity change over 6-month follow-up**

|  | **Decreased LPA over 6-months follow-up** | **Increased LPA over 6-month follow-up** | **Sig_._** |
| --- | --- | --- | --- |
| Subjects n | 45 | 54 |  |
| Demographic and anthropometric data | | | |
| Age (years) mean±SD | 55±16 | 56±15 | p=0.66 |
| Male n(%) | 25(56) | 25(46) | p=0.42 |
| BMI (kg·m^-2^) mean±SD | 28.2±5.1 | 27.3±5.0 | p=0.38 |
| Lung function | | | |
| FEV_1_%predicted mean±SD | 91±14 | 89±15 | p=0.90 |
| FVC%predicted mean±SD | 87±13 | 87±13 | p=0.60 |
| Physical activity | | | |
| LPA (min·day^-1^) mean±SD | 331±133 | 238±107 | p<0.01* |
| MVPA (min·day^-1^) mean±SD | 42±31 | 59±51 | p=0.04* |
| Steps·day^-1^ mean±SD | 5676±3621 | 5349±3087 | p=0.57 |
| Steps·day^-1^≤5000 n(%). | 23(51) | 26(48) | p=0.84 |
| Sedentary time (min·day^-1^) mean±SD | 433±140 | 408±143 | p=0.42 |
| Functional capacity | | | |
| 6MWD (m) mean±SD | 587±131 | 576±114 | p=0.94 |
| 6MWD≤70%predicted n(%) | 11(24) | 5(9) | p=0.06 |
| 1minSTS (reps) mean±SD | 27±11 | 26±79 | p=0.61 |
| 1minSTS≤70%predicted n(%) | 12(27) | 17(32) | p=0.66 |
| Peripheral muscle strength | | | |
| QMVC (kgf) mean±SD | 18±6 | 16±5 | p=0.34 |
| QMVC≤70%predicted n(%). | 8(18) | 8(15) | p=0.79 |
| Symptoms median(Q1;Q3) | 2(0;3) | 1(0;2) | p=0.19 |
| Long COVID n(%) | 31(69) | 37(69) | p=1.00 |
| Dyspnoea mMRC median(Q1;Q3) | 1(0;1) | 0(0;1) | p=0.08 |
| mMRC≥2 n(%). | 8(18) | 2(4) | p=0.04* |
| Fatigue FACIT-FS median(Q1;Q3) | 42(32;49) | 41(35;49) | p=1.00 |
| FACIT-FS≤43 n(%). | 26(58) | 32(59) | p=1.00 |
| Anxiety HADS-A median(Q1;Q3) | 6(3;9) | 4;2;6) | p=0.06 |
| HADS-A≥8 n(%). | 20(44) | 11(20) | p=0.02* |
| Depression HADS-D median(Q1;Q3) | 4(3;6) | 4(3;6) | p=0.77 |
| HADS-D≥8 n(%). | 11(24) | 10(19) | p=0.62 |
| Health-related quality of life | | | |
| EQ-5D-5L median(Q1;Q3) | 80(60;85) | 80(70;90) | p=0.22 |
| EQ-5D-5L≤74/female or 78/male n(%). | 20(44) | 20(37) | p=0.54 |
| Abbreviations. 1minSTS: 1-min sit-to-stand test; 6MWD: 6-min walking distance; BMI: body mass index; EQ-5D-5L: European quality of life - 5 dimensions - 5 levels; FACIT-FS: functional assessment of chronic illness therapy - fatigue; FEV_1_: forced expiratory volume in 1s; FVC: forced vital capacity; HADS: hospital anxiety and depression scale; LPA: time spent in light physical activity; mMRC: modified Medical Research Council; MVPA: time spent in moderate-to-vigorous physical activity; QMVC: quadriceps maximal voluntary contraction.  *Statistically significant p-value <0.05 between groups | | | |

### **Table S2. Correlations between time spent in light physical activity at eighteen-months and all baseline potential predictors**

| Dependent | Predictors | r | ρ | 95%CI ll | 95%CI ul | Sig. |
| --- | --- | --- | --- | --- | --- | --- |
| LPA at eighteen-months (min·day^-1^) | Age (years) | 0.15 |  | -0.05 | 0.34 | p=0.13 |
|  | BMI (kg·m^-2^) | -0.09 |  | -0.28 | 0.11 | p=0.36 |
|  | FEV_1_%predicted | 0.08 |  | -0.12 | 0.28 | p=0.42 |
|  | FVC%predicted | 0.13 |  | -0.08 | 0.32 | p=0.22 |
|  | LPA at baseline (min·day^-1^) | 0.47 |  | 0.30 | 0.61 | p<0.01* |
|  | MVPA at baseline (min·day^-1^) | 0.11 |  | -0.09 | 0.30 | p=0.27 |
|  | Steps·day^-1^ baseline | 0.23 |  | 0.03 | 0.41 | p=0.02* |
|  | Sedentary time at baseline (min·day^-1^) | -0.36 |  | -0.52 | -0.18 | p<0.01* |
|  | 6MWD (m) | -0.04 |  | -0.24 | 0.16 | p=0.69 |
|  | 1minSTS (reps) | -0.01 |  | -0.20 | 0.20 | p=0.99 |
|  | QMVC (kgf) | -0.07 |  | -0.27 | 0.13 | p=0.47 |
|  | Symptoms n |  | -0.04 | -0.24 | 0.17 | p=0.70 |
|  | Dyspnoea mMRC |  | -0.10 | -0.29 | 0.11 | p=0.35 |
|  | Fatigue FACIT-FS |  | 0.03 | -0.17 | 0.24 | p=0.72 |
|  | Anxiety HADS-A |  | -0.02 | -0.22 | 0.18 | p=0.85 |
|  | Depression HADS-D |  | 0.06 | -0.15 | 0.26 | p=0.56 |
|  | EQ-5D-5L |  | 0.09 | -0.12 | 0.29 | p=0.39 |
| Abbreviations. 1minSTS: 1-min sit-to-stand test; 6MWD: 6-min walking distance; BMI: body mass index; EQ-5D-5L: European quality of life - 5 dimensions - 5 levels; FACIT-FS: functional assessment of chronic illness therapy - fatigue; FEV_1_: forced expiratory volume in 1s; FVC: forced vital capacity; HADS: hospital anxiety and depression scale; LPA; time spent in light physical activity; mMRC: modified Medical Research Council; MVPA: time spent in moderate-to-vigorous physical activity; QMVC: quadriceps maximal voluntary contraction.  *Statistically significant p-value <0.05 | | | | | | |

### **Table S3. Potential predictors for time spent in light physical activity eighteen-months, starting at twelve-months post-COVID-19 - Stepwise regression method vs. LASSO regression method**

| Stepwise regression model | | | | | | | |  |
| --- | --- | --- | --- | --- | --- | --- | --- | --- |
| Dependent | **Predictors** | **β** | | **95%CI** | | **p-value** | |  |
| LPA (min·day^-1^) eighteen-months | (Intercept) | 330.10 | | 241.41;418.9 | | 9.80e^-11^* | |  |
|  | LPA (min·day^-1^) | 0.32 | | 0.18;0.46 | | 3.18e^-5^* | |  |
|  | Sedentary time (min·day^-1^) | -0.20 | | -0.34;-0.06 | | 0.0035* | |  |
|  | Dyspnoea mMRC≥2 | -70.17 | | -129.17;-11.17 | | 0.0219* | |  |
| R^2^ = 0.35  Adjusted R^2^ = 0.32  After bootstrapping  Adjusted R^2^ = 0.32  Adjusted calibration slope = 0.95  Adjusted calibration intercept = 13.44  F(4.93) = 12.52, p<0.001  Tolerance = 0.92-0.96; VIF = 1.09-1.04  Eigenvalue between 3.76 and 0.03; condition index between 1.00 and 11.48 | | | | | | | |  |
| LASSO regression model | | | | | | | | |
| Dependent | Predictors | | β | | 95%CI | | p-value | |
| LPA (min·day^-1^) eighteen-months | (Intercept) | | 388.44 | | 243.93;532.95 | | <0.0001* | |
|  | Dyspnoea mMRC | | -21.16 | | -61.87;19.55 | | 0.3110 | |
|  | Dyspnoea mMRC≥2 | | -41.65 | | -129.30;46.00 | | 0.3542 | |
|  | Fatigue FACIT-F | | -1.77 | | -4.20;0.66 | | 0.1575 | |
|  | Anxiety HADS-A≥7 | | -33.95 | | -76.30;8.40 | | 0.12 | |
|  | Sedentary time (min·day^-1^) | | -0.18 | | -0.34;-0.02 | | 0.0171* | |
|  | LPA (min·day^-1^) | | 0.35 | | 0.17;0.53 | | 0.0001* | |
|  | MVPA (min·day^-1^) | | 0.29 | | -0.20;0.78 | | 0.2488 | |
|  | Steps·day^-1^ | | -0.00 | | -0.00;0.00 | | 0.9686 | |
| R^2^ = 0.39; λ = 1.23  Adjusted R^2^ = 0.32  Adjusted calibration slope = 0.89  Adjusted calibration intercept = 31.30  F(9, 88) = 6.11, p < 0.001 | | | | | | | | |
| *Statistically significant p-value<0.05  Abbreviations: FACIT-F: functional assessment of chronic illness therapy; HADS: hospital anxiety and depression scale; LPA: time spent in light physical activity; mMRC: modified Medical Research Council; MVPA: time spent in moderate-to-vigorous physical activity; VIF: variance inflation factor. | | | | | | | | |

### **Table S4. People after COVID-19 at twelve-months post-discharge, grouped by time spent in moderate-to-vigorous change over 6-month follow-up**

|  | **Decreased MVPA over 6-months follow-up** | **Increased MVPA over 6-month follow-up** | **Sig_._** |
| --- | --- | --- | --- |
| Subjects n | 55 | 44 |  |
| Demographic and anthropometric data | | | |
| Age (years) mean±SD | 57±14 | 54±16 | p=0.41 |
| Male n(%) | 30(55) | 20(46) | p=0.42 |
| BMI (kg·m^-2^) mean±SD | 27.6±4.8 | 28.0±5.3 | p=0.72 |
| Lung function | | | |
| FEV_1_%predicted mean±SD | 91±13 | 89±16 | p=0.80 |
| FVC%predicted mean±SD | 88±11 | 86±15 | p=0.75 |
| Physical activity | | | |
| LPA (min·day^-1^) mean±SD | 252±134 | 313±111 | p=0.02* |
| MVPA (min·day^-1^) mean±SD | 71±49 | 29±19 | p<0.01* |
| Steps·day^-1^ mean±SD | 6246±3617 | 4589±2694 | p=0.01* |
| Steps·day^-1^≤5000 n(%). | 25(46) | 24(55) | p=0.42 |
| Sedentary time (min·day^-1^) mean±SD | 423±153 | 414±127 | p=0.81 |
| Functional capacity | | | |
| 6MWD (m) mean±SD | 574±120 | 589±123 | p=0.38 |
| 6MWD≤70%predicted n(%) | 11(20) | 5(11) | p=0.28 |
| 1minSTS (reps) mean±SD | 27±11 | 26±9 | p=0.75 |
| 1minSTS≤70%predicted n(%) | 13(24) | 16(36) | p=0.18 |
| Peripheral muscle strength | | | |
| QMVC (kgf) mean±SD | 17±5 | 16±5 | p=0.50 |
| QMVC≤70%predicted n(%). | 7(13) | 9(21) | p=0.41 |
| Symptoms median(Q1;Q3) | 2(1;3) | 1(0;3) | p=0.15 |
| Long COVID n(%) | 42(76) | 26(59) | p=0.08 |
| Dyspnoea mMRC median(Q1;Q3) | 1(0;1) | 1(0;1) | p=0.90 |
| mMRC≥2 n(%). | 4(7) | 6(14) | p=0.33 |
| Fatigue FACIT-FS median(Q1;Q3) | 39(35;48) | 44(37;50) | p=0.17 |
| FACIT-FS≤43 n(%). | 36(66) | 22(50) | p=0.15 |
| Anxiety HADS-A median(Q1;Q3) | 4(3;8) | 5(2;7) | p=0.83 |
| HADS-A≥8 n(%). | 17(31) | 14(32) | p=1.00 |
| Depression HADS-D median(Q1;Q3) | 4(3;7) | 4(3;6) | p=0.31 |
| HADS-D≥8 n(%). | 15(27) | 6(14) | p=0.14 |
| Health-related quality of life | | | |
| EQ-5D-5L median(Q1;Q3) | 75(63;88) | 85(70;94) | p=0.04* |
| EQ-5D-5L≤74/female or 78/male n(%). | 26(47) | 14(32) | p=0.14 |
| Abbreviations. 1minSTS: 1-min sit-to-stand test; 6MWD: 6-min walking distance; BMI: body mass index; EQ-5D-5L: European quality of life - 5 dimensions - 5 levels; FACIT-FS: functional assessment of chronic illness therapy - fatigue; FEV_1_: forced expiratory volume in 1s; FVC: forced vital capacity; HADS: hospital anxiety and depression scale; LPA: time spent in light physical activity; mMRC: modified Medical Research Council; MVPA: time spent in moderate-to-vigorous physical activity; QMVC: quadriceps maximal voluntary contraction.  *Statistically significant p-value <0.05 between groups | | | |

### **Table S5. Correlations between time spent in moderate-to-vigorous physical activity at eighteen-months and all baseline potential predictors**

| Dependent | Predictors | r | ρ | 95%CI ll | 95%CI ul | Sig. |
| --- | --- | --- | --- | --- | --- | --- |
| MVPA at eighteen-months (min·day^-1^) | Age (years) | -0.23 |  | -0.41 | -0.03 | p=0.02* |
|  | BMI (kg·m^-2^) | 0.11 |  | -0.09 | 0.30 | p=0.30 |
|  | FEV_1_%predicted | 0.08 |  | -0.13 | 0.27 | p=0.46 |
|  | FVC%predicted | 0.02 |  | -0.18 | 0.22 | p=0.84 |
|  | LPA at baseline (min·day^-1^) | 0.09 |  | -0.11 | 0.29 | p=0.40 |
|  | MVPA at baseline (min·day^-1^) | 0.18 |  | -0.02 | 0.36 | p=0.08 |
|  | Steps·day^-1^ baseline | 0.11 |  | -0.10 | 0.30 | p=0.30 |
|  | Sedentary time at baseline (min·day^-1^) | -0.27 |  | -0.44 | -0.07 | p<0.01* |
|  | 6MWD (m) | 0.14 |  | -0.06 | 0.33 | p=0.17 |
|  | 1minSTS (reps) | 0.20 |  | 0.00 | 0.38 | p=0.04* |
|  | QMVC (kgf) | 0.09 |  | -0.11 | 0.29 | p=0.35 |
|  | Symptoms n |  | -0.08 | -0.28 | 0.13 | p=0.45 |
|  | Dyspnoea mMRC |  | -0.11 | -0.30 | 0.10 | p=0.29 |
|  | Fatigue FACIT-FS |  | 0.06 | -0.15 | 0.26 | p=0.56 |
|  | Anxiety HADS-A |  | 0.16 | -0.04 | 0.36 | p=0.11 |
|  | Depression HADS-D |  | 0.04 | -0.17 | 0.24 | p=0.71 |
|  | EQ-5D-5L |  | 0.10 | -0.11 | 0.29 | p=0.35 |
| Abbreviations. 1minSTS: 1-min sit-to-stand test; 6MWD: 6-min walking distance; BMI: body mass index; EQ-5D-5L: European quality of life - 5 dimensions - 5 levels; FACIT-FS: functional assessment of chronic illness therapy - fatigue; FEV_1_: forced expiratory volume in 1s; FVC: forced vital capacity; HADS: hospital anxiety and depression scale; LPA: time spent in light physical activity; mMRC: modified Medical Research Council; MVPA: time spent in moderate-to-vigorous physical activity; QMVC: quadriceps maximal voluntary contraction.  *Statistically significant p-value <0.05 | | | | | | |

### **Table S6. Potential predictors for time spent in moderate-to-vigorous physical activity at eighteen-months, starting at twelve-months post-COVID-19 - Stepwise regression method vs. LASSO regression method**

| Stepwise regression model | | | | | | | | |
| --- | --- | --- | --- | --- | --- | --- | --- | --- |
| Dependent | **Predictors** | | **β** | | **95%CI** | | **p-value** | |
| MVPA (min·day^-1^) eighteen-months | (Intercept) | | 46.14 | | 26.14, 66.14 | | 1.78e^-5^ | |
|  | Sedentary time (min·day^-1^) | | -0.05 | | -0.09;-0.01 | | 0.0061* | |
|  | 1minSTS (reps) | | 0.41 | | -0.08;0.90 | | 0.1085 | |
| R^2^ = 0.11  Adjusted R^2^ = 0.09  After bootstrapping  Adjusted R^2^ = 0.09  Adjusted calibration slope = 0.99  Adjusted calibration intercept = 0.29  F(2, 95) = 5.79, p = 0.0042  Tolerance = 0.98; VIF = 1.01  Eigenvalue between 2.85 and 0.03; condition index between 1.00 and 9.21 | | | | | | | | |
| LASSO regression model | | | | | | | | |
| Dependent | | **Predictors** | | **β** | | **95%CI** | | **p-value** |
| MVPA (min·day^-1^) eighteen-months | | (Intercept) | | 46.14 | | 26.14;66.14 | | <0.0001* |
|  | | 1minSTS (reps) | | 0.41 | | -0.08;0.90 | | 0.1085 |
|  | | Sedentary time (min·day^-1^) | | -0.05 | | -0.09;-0.01 | | 0.0061* |
| R^2^ = 0.11; λ = 3.28  Adjusted R^2^ = 0.09  Adjusted calibration slope = 0.97  Adjusted calibration intercept = 0.94  F(2, 95) = 5.79, p = 0.0042 | | | | | | | | |
| *Statistically significant p-value<0.05  Abbreviations: 1minSTS: 1-minute sit-to-stand test; VIF: variance inflation factor. | | | | | | | | |

### **Table S7. People after COVID-19 at twelve-months post-discharge, grouped by steps per day change over 6-month follow-up**

|  | **Decreased steps/day over 6-months follow-up** | **Increased steps/day over 6-month follow-up** | **Sig_._** |
| --- | --- | --- | --- |
| Subjects n | 60 | 39 |  |
| Demographic and anthropometric data | | | |
| Age (years) mean±SD | 55±16 | 57±14 | p=0.40 |
| Male n(%) | 29(48) | 21(54) | p=0.68 |
| BMI (kg·m^-2^) mean±SD | 26.7±4.5 | 29.3±5.4 | p=0.02* |
| Lung function | | | |
| FEV_1_%predicted mean±SD | 91±13 | 88±16 | p=0.16 |
| FVC%predicted mean±SD | 88±12 | 86±15 | p=0.24 |
| Physical activity | | | |
| LPA (min·day^-1^) mean±SD | 299±127 | 249±123 | p=0.03* |
| MVPA (min·day^-1^) mean±SD | 54±44 | 47±43 | p=0.44 |
| Steps·day^-1^ mean±SD | 6702±3196 | 3618±2580 | p<0.01* |
| Steps·day^-1^≤5000 n(%). | 18(30) | 31(80) | p<0.01* |
| Sedentary time (min·day^-1^) mean±SD | 424±143 | 412±141 | p=0.85 |
| Functional capacity | | | |
| 6MWD (m) mean±SD | 592±111 | 564±135 | p=0.22 |
| 6MWD≤70%predicted n(%) | 9(15) | 7(18) | p=0.78 |
| 1minSTS (reps) mean±SD | 28±11 | 24±8 | p=0.07 |
| 1minSTS≤70%predicted n(%) | 18(30) | 11(28) | p=1.00 |
| Peripheral muscle strength | | | |
| QMVC (kgf) mean±SD | 17±5 | 16±5 | p=0.21 |
| QMVC≤70%predicted n(%). | 5(8) | 11(28) | p=0.01* |
| Symptoms median(Q1;Q3) | 1(0;3) | 1(0;3) | p=0.66 |
| Long COVID n(%) | 40(67) | 28(72) | p=0.66 |
| Dyspnoea mMRC median(Q1;Q3) | 0(0;1) | 1(0;1) | p=0.31 |
| mMRC≥2 n(%). | 5(8) | 5(13) | p=0.51 |
| Fatigue FACIT-FS median(Q1;Q3) | 42(36;49) | 39(32;48) | p=0.20 |
| FACIT-FS≤43 n(%). | 34(57) | 24(62) | p=0.68 |
| Anxiety HADS-A median(Q1;Q3) | 5(3;8) | 4(2;6) | p=0.63 |
| HADS-A≥8 n(%). | 21(35) | 10(26) | p=0.38 |
| Depression HADS-D median(Q1;Q3) | 4(3;6) | 4(3;6) | p=0.55 |
| HADS-D≥8 n(%). | 12(20) | 9(23) | p=0.80 |
| Health-related quality of life | | | |
| EQ-5D-5L median(Q1;Q3) | 85(70;90) | 77(60;85) | p=0.11 |
| EQ-5D-5L≤74/female or 78/male n(%). | 22(37) | 18(46) | p=0.40 |
| Abbreviations. 1minSTS: 1-min sit-to-stand test; 6MWD: 6-min walking distance; BMI: body mass index; EQ-5D-5L: European quality of life - 5 dimensions - 5 levels; FACIT-FS: functional assessment of chronic illness therapy - fatigue; FEV_1_: forced expiratory volume in 1s; FVC: forced vital capacity; HADS: hospital anxiety and depression scale; LPA: time spent in light physical activity; mMRC: modified Medical Research Council; MVPA: time spent in moderate-to-vigorous physical activity; QMVC: quadriceps maximal voluntary contraction.  *Statistically significant p-value <0.05 between groups | | | |

### **Table S8. Correlations between steps per day at eighteen-months and all baseline potential predictors**

| Dependent | Predictors | r | ρ | 95%CI ll | 95%CI ul | Sig. |
| --- | --- | --- | --- | --- | --- | --- |
| Steps·day^-1^ eighteen-months | Age (years) | -0.12 |  | -0.31 | 0.08 | p=0.23 |
|  | BMI (kg·m^-2^) | -0.07 |  | -0.26 | 0.13 | p=0.49 |
|  | FEV_1_%predicted | 0.34 |  | 0.15 | 0.50 | p<0.01* |
|  | FVC%predicted | 0.32 |  | 0.13 | 0.49 | p<0.01* |
|  | LPA at baseline (min·day^-1^) | 0.20 |  | 0.00 | 0.38 | p=0.04* |
|  | MVPA at baseline (min·day^-1^) | 0.07 |  | -0.13 | 0.27 | p=0.48 |
|  | Steps·day^-1^ baseline | 0.53 |  | 0.37 | 0.66 | p<0.01* |
|  | Sedentary time at baseline (min·day^-1^) | -0.12 |  | -0.31 | 0.08 | p=0.22 |
|  | 6MWD (m) | 0.27 |  | 0.08 | 0.44 | p<0.01* |
|  | 1minSTS (reps) | 0.19 |  | -0.01 | 0.38 | p=0.06 |
|  | QMVC (kgf) | 0.12 |  | -0.08 | 0.31 | p=0.23 |
|  | Symptoms n |  | -0.14 | -0.34 | 0.06 | p=0.15 |
|  | Dyspnoea mMRC |  | -0.23 | -0.41 | -0.03 | p=0.02* |
|  | Fatigue FACIT-FS |  | 0.13 | -0.07 | 0.33 | p=0.20 |
|  | Anxiety HADS-A |  | 0.05 | -0.16 | 0.25 | p=0.66 |
|  | Depression HADS-D |  | -0.03 | -0.23 | 0.18 | p=0.79 |
|  | EQ-5D-5L |  | 0.09 | -0.12 | 0.28 | p=0.40 |
| Abbreviations. 1minSTS: 1-min sit-to-stand test; 6MWD: 6-min walking distance; BMI: body mass index; EQ-5D-5L: European quality of life - 5 dimensions - 5 levels; FACIT-FS: functional assessment of chronic illness therapy - fatigue; FEV_1_: forced expiratory volume in 1s; FVC: forced vital capacity; HADS: hospital anxiety and depression scale; LPA: time spent in light physical activity; mMRC: modified Medical Research Council; MVPA: time spent in moderate-to-vigorous physical activity; QMVC: quadriceps maximal voluntary contraction.  *Statistically significant p-value <0.05 | | | | | | |

### **Table S9. Potential predictors for steps per day at eighteen-months, starting at twelve-months post-COVID-19 - Stepwise regression method vs. LASSO regression method**

| Stepwise regression model | | | | | | | |
| --- | --- | --- | --- | --- | --- | --- | --- |
| Dependent | **Predictors** | **β** | | **95%CI** | | **p-value** | |
| Steps·day^-1^ eighteen-months | (Intercept) | -1463.43 | | -4442.83;1515.97 | | 0.3366 | |
|  | Steps·day^-1^ | 0.39 | | 0.23;0.55 | | 1.16e^-6^* | |
|  | FEV_1_%predicted | 31.28 | | -2.50;65.06 | | 0.0729 | |
| R^2^ = 0.34  Adjusted R^2^ = 0.31  After bootstrapping  Adjusted R^2^ = 0.31  Adjusted calibration slope = 0.97  Adjusted calibration intercept = 152.11  F(3, 94) = 15.78, p = 2.18e^-8^  Tolerance = 0.89-0.96; VIF = 1.12-1.05  Eigenvalue between 3.70 and 0.01; condition index between 1.00 and 16.49 | | | | | | | |
| LASSO regression model | | | | | | |  |
| Dependent | **Predictors** | **β** | **95%CI** | | **p-value** | |  |
| Steps·day^-1^ eighteen-months | (Intercept) | -5087.56 | -10414.99;239.87 | | 0.0642 | |  |
|  | BMI (kg·m^-2^) | 106.79 | -7.74;221.32 | | 0.0710 | |  |
|  | FEV_1_%predicted | 34.46 | 0.04;68.88 | | 0.0530 | |  |
|  | 6MWD (m) | 1.37 | -4.02;6.76 | | 0.6195 | |  |
|  | QMVC≤70%predicted | -1251.39 | -2661.69; 158.91 | | 0.0856 | |  |
|  | Dyspnoea mMRC | -416.13 | -1223.83;391.57 | | 0.3151 | |  |
|  | Steps·day^-1^ | 0.44 | 0.28;0.60 | | <0.0001* | |  |
| R^2^ = 0.37; λ = 125.44  Adjusted R^2^ = 0.32  Adjusted calibration slope = 0.91  Adjusted calibration intercept = 449.45  F(7, 90) = 7.62, p < 0.0001 | | | | | | |  |
| *Statistically significant p-value<0.05  Abbreviations: 6MWD: 6-minute walking distance; BMI: body mass index; FEV_1_%predicted: forced expiratory volume in one second ; mMRC: modified Medical Research Council; QMVC: quadriceps maximal voluntary contraction; VIF: variance inflation factor | | | | | | |  |

### **Table S10. People after COVID-19 at twelve-months post-discharge, grouped by sedentary time change over 6-month follow-up**

|  | **Decreased sedentary time over 6-months follow-up** | **Increased sedentary time over 6-month follow-up** | **Sig_._** |
| --- | --- | --- | --- |
| Subjects n | 53 | 46 |  |
| Demographic and anthropometric data | | | |
| Age (years) mean±SD | 56±15 | 55±16 | p=0.82 |
| Male n(%) | 28(53) | 22(48) | p=0.69 |
| BMI (kg·m^-2^) mean±SD | 27.6±5.3 | 28.0±4.7 | p=0.68 |
| Lung function | | | |
| FEV_1_%predicted mean±SD | 91±15 | 89±14 | p=0.39 |
| FVC%predicted mean±SD | 88±14 | 87±12 | p=0.51 |
| Physical activity | | | |
| LPA (min·day^-1^) mean±SD | 287±127 | 270±129 | p=0.54 |
| MVPA (min·day^-1^) mean±SD | 45±32 | 59±54 | p=0.13 |
| Steps·day^-1^ mean±SD | 5655±3449 | 5300±3187 | p=0.71 |
| Steps·day^-1^≤5000 n(%). | 26(48) | 23(50) | p=1.00 |
| Sedentary time (min·day^-1^) mean±SD | 473±152 | 354±93 | p<0.01* |
| Functional capacity | | | |
| 6MWD (m) mean±SD | 591±111 | 569±132 | p=0.21 |
| 6MWD≤70%predicted n(%) | 6(11) | 10(22) | p=0.18 |
| 1minSTS (reps) mean±SD | 26±9 | 27±11 | p=0.70 |
| 1minSTS≤70%predicted n(%) | 14(26) | 15(33) | p=0.52 |
| Peripheral muscle strength | | | |
| QMVC (kgf) mean±SD | 17±5 | 17±5 | p=0.90 |
| QMVC≤70%predicted n(%). | 10(19) | 6(13) | p=0.59 |
| Symptoms median(Q1;Q3) | 1(0;3) | 2(0;3) | p=0.20 |
| Long COVID n(%) | 35(66) | 33(72) | p=0.67 |
| Dyspnoea mMRC median(Q1;Q3) | 1)0;1) | 1(0;1) | p=0.40 |
| mMRC≥2 n(%). | 2(4) | 8(17) | p=0.04* |
| Fatigue FACIT-FS median(Q1;Q3) | 42(36;50) | 41(34;47) | p=0.45 |
| FACIT-FS≤43 n(%). | 29(55) | 29(63) | p=0.42 |
| Anxiety HADS-A median(Q1;Q3) | 4(2;8) | 5(3;7) | p=0.39 |
| HADS-A≥8 n(%). | 15(28) | 16(35) | p=0.52 |
| Depression HADS-D median(Q1;Q3) | 3(3;6) | 5(3;6) | p=0.04* |
| HADS-D≥8 n(%). | 7(13) | 14(30) | p=0.04* |
| Health-related quality of life | | | |
| EQ-5D-5L median(Q1;Q3) | 80(70;90) | 80(61;89) | p=0.21 |
| EQ-5D-5L≤74/female or 78/male n(%). | 20(38) | 20(44) | p=0.68 |
| Abbreviations. 1minSTS: 1-min sit-to-stand test; 6MWD: 6-min walking distance; BMI: body mass index; EQ-5D-5L: European quality of life - 5 dimensions - 5 levels; FACIT-FS: functional assessment of chronic illness therapy - fatigue; FEV_1_: forced expiratory volume in 1s; FVC: forced vital capacity; HADS: hospital anxiety and depression scale; LPA: time spent in light physical activity; mMRC: modified Medical Research Council; MVPA: time spent in moderate-to-vigorous physical activity; QMVC: quadriceps maximal voluntary contraction.  *Statistically significant p-value <0.05 between groups | | | |

### **Table S11. Correlations between sedentary time at eighteen-months and all baseline potential predictors**

| Dependent | Predictors | r | ρ | 95%CI ll | 95%CI ul | Sig. |
| --- | --- | --- | --- | --- | --- | --- |
| Sedentary time at eighteen-months (min·day^-1^) | Age (years) | 0.07 |  | -0.13 | 0.26 | p=0.51 |
|  | BMI (kg·m^-2^) | 0.06 |  | -0.14 | 0.25 | p=0.58 |
|  | FEV_1_%predicted | -0.12 |  | -0.31 | 0.08 | p=0.24 |
|  | FVC%predicted | -0.15 |  | -0.34 | 0.05 | p=0.14 |
|  | LPA at baseline (min·day^-1^) | -0.40 |  | -0.55 | -0.22 | p<0.01* |
|  | MVPA at baseline (min·day^-1^) | -0.16 |  | -0.34 | 0.04 | p=0.13 |
|  | Steps·day^-1^ baseline | -0.28 |  | -0.45 | -0.01 | p=0.01* |
|  | Sedentary time at baseline (min·day^-1^) | 0.35 |  | 0.16 | 0.51 | p<0.01* |
|  | 6MWD (m) | -0.01 |  | -0.21 | 0.19 | p=0.94 |
|  | 1minSTS (reps) | -0.05 |  | -0.25 | 0.15 | p=0.59 |
|  | QMVC (kgf) | -0.03 |  | -0.22 | 0.17 | p=0.79 |
|  | Symptoms n |  | 0.04 | -0.16 | 0.24 | p=0.68 |
|  | Dyspnoea mMRC |  | 0.16 | -0.04 | 0.36 | p=0.10 |
|  | Fatigue FACIT-FS |  | -0.08 | -0.28 | 0.13 | p=0.43 |
|  | Anxiety HADS-A |  | -0.08 | -0.28 | 0.13 | p=0.43 |
|  | Depression HADS-D |  | 0.01 | -0.19 | 0.22 | p=0.90 |
|  | EQ-5D-5L |  | -0.16 | -0.35 | 0.04 | p=0.11 |
| Abbreviations. 1minSTS: 1-min sit-to-stand test; 6MWD: 6-min walking distance; BMI: body mass index; EQ-5D-5L: European quality of life - 5 dimensions - 5 levels; FACIT-FS: functional assessment of chronic illness therapy - fatigue; FEV_1_: forced expiratory volume in 1s; FVC: forced vital capacity; HADS: hospital anxiety and depression scale; LPA: time spent in light physical activity; mMRC: modified Medical Research Council; MVPA: time spent in moderate-to-vigorous physical activity; QMVC: quadriceps maximal voluntary contraction.  *Statistically significant p-value <0.05 | | | | | | |

### **Table S12. Potential predictors for sedentary time at eighteen-months, starting at twelve-months post-COVID-19 - Stepwise regression method vs. LASSO regression method**

| Stepwise regression model | | | | |  |
| --- | --- | --- | --- | --- | --- |
| Dependent | **Predictors** | **β** | **95%CI** | **p-value** |  |
| Sedentary time (min·day^-1^) eighteen-months | (Intercept) | 233.32 | 42.37;424.27 | 0.0186* |  |
|  | LPA (min·day^-1^) | -0.39 | -0.63;-0.16 | 0.0009* |  |
|  | Sedentary time (min·day^-1^) | 0.30 | 0.08;0.52 | 0.0052* |  |
|  | Dyspnoea mMRC | 62.35 | 16.17;108.53 | 0.0096* |  |
|  | Fatigue FACIT-FS | 2.62 | -0.87;6.11 | 0.1450 |  |
| R^2^ = 0.28  Adjusted R^2^ = 0.25  F(4;93) = 9.26, p = 2.42e^-6^  After bootstrapping  Adjusted R^2^ = 0.25  Adjusted calibration slope = 0.96  Adjusted calibration intercept = 16.33  F(4,94) = 9.26 p = 2.36e^-6^  Tolerance = 0.91-0.71; VIF = 1.10-1.41  Eigenvalue between 4.22 and 0.02; condition index between 1.00 and 16.97 | | | | |  |
| LASSO regression model | | | | |  |
| Dependent | **Predictors** | **β** | **95%CI** | **p-value** | |
| Sedentary time (min·day^-1^) eighteen-months | (Intercept) | 361.50 | 232.34;490.66 | <0.001* | |
|  | Dyspnoea mMRC | 24.10 | -34.60;82.80 | 0.4229 | |
|  | Dyspnoea mMRC≥2 | 47.21 | -90.57;184.99 | 0.5022 | |
|  | Sedentary time (min·day^-1^) | 0.32 | 0.10;0.54 | 0.0036 | |
|  | LPA (min·day^-1^) | -0.34 | -0.58;-0.11 | 0.0082 | |
|  | Steps·day^-1^ | -0.01 | -0.03;0.01 | 0.2991 | |
| R^2^ = 0.25; λ = 15.87  Adjusted R^2^ = 0.24  Adjusted calibration slope = 0.92  Adjusted calibration intercept = 32.44  F(5,92) = 7.22, p < 0.001 | | | | | |
| *Statistically significant p-value<0.05  Abbreviations: FACIT-FS: functional assessment of chronic illness therapy-fatigue; LPA: time spent in light physical activity; mMRC: modified Medical Research Council; VIF: variance inflation factor. | | | | | |

### **Table S13. Participants’ characteristics at twelve-months after COVID-19, exploratory analyses**

|  | **All** | **H group** | **HW group** | **ICU group** |
| --- | --- | --- | --- | --- |
| **Participants n** | 148 | 44 | 60 | 44 |
| **Demographic and anthropometric data** | | | | |
| Age (years) mean±SD | 58±15 | 48±15* | 63±14 | 60±12 |
| Male | 80(54) | 16(36)* | 35(58) | 29(66) |
| BMI (kg·m^-2^) mean±SE | 28.2±0.4 | 26.0±0.7* | 28.6±0.6 | 30.1±0.7 |
| **Lung function** | | | | |
| FEV_1_%predicted mean±SE | 89±1 | 95±2 | 89±2 | 83±2 |
| FVC%predicted mean±SE | 85±1 | 92±2* | 83±2 | 80±2 |
| **Medical records** | | | | |
| **Comorbidities median(Q1;Q3)** | **2(1;3)** | **1(0;2)*** | **2(1;3)** | **2(1;3)** |
| Endocrine or metabolic diseases^¶^ n(%) | 60(41) | 11(25)* | 26(43) | 23(52) |
| Circularity system diseases^¶^ n(%) | 59(40) | 6(14)* | 32(53) | 21(48) |
| Musculoskeletal diseases^¶^ n(%) | 30(20) | 10(23) | 11(18) | 9(21) |
| Respiratory system diseases^¶^ n(%) | 22(15) | 7(16) | 11(18) | 4(9) |
| Immune system diseases^¶^ n(%) | 17(12) | 5(11) | 11(18) | 1(2) |
| Nervous system diseases^¶^ n(%) | 12(8) | 4(9) | 2(3) | 6(14) |
| Others^¶^ n(%) | 33(22) | 5(11) | 20(33) | 8(18) |
| Charlson comorbidity index median(Q1;Q3) | 2(0;3) | 0(0;2)* | 3(1;4) | 2(1;3) |
| Charlson comorbidity categories (mild/moderate/severe) n(%) | 53(36)/48(32)/8(5) | 15(34)/6(14)/0(0)* | 20(33)/26(43)/5(8) | 18(41)/16(36)/3(7) |
| **Medications median(Q1;Q3)** | **1(0;2)** | **1(0;1)*** | **1(1;3)** | **1(1;3)** |
| Cardiovascular system^§^ n(%) | 65(44) | 8(18)* | 35(58) | 22(50) |
| Alimentary tract and metabolism^§^ n(%) | 25(17) | 2(5)* | 11(18) | 12(27) |
| Respiratory system^§^ n(%) | 20(14) | 5(11) | 11(18) | 4(9) |
| Systemic hormonal preparations, and insulins^§^ n(%) | 19(13) | 7(16) | 7(12) | 5(11) |
| Nervous system^§^ n(%) | 15(10) | 4(9) | 7(12) | 4(9) |
| Others^§^ n(%) | 13 (9) | 0(0) | 9(15) | 4(9) |
| All data, reported as mean±SE, are adjusted for age and sex.  Abbreviations. BMI: body mass index; FEV_1_: forced expiratory volume in 1s; FVC: forced vital capacity; H: home; HW: hospital ward; ICU: intensive care unit.  ^#^Missing values, as follows. Intention-to-treat analysis set: Lung function (n=2).  *Statistically significant p-value <0.05 between groups.  ¶International Classification of Diseases, 11^th^ Ed. Others includes: infectious diseases, ear disorders, digestive system disorders, genitourinary system diseases, developmental anomalies, sleep-awake disorders, mental disorders and symptoms and signs not elsewhere classified.  #Anatomical Therapeutic Chemical Classification System. Others includes: dermatologicals, musculoskeletal system, and blood and blood forming organs. | | | | |

### **Table S14. Primary and secondary outcomes in people after COVID-19, at twelve, fifteen and eighteen months after discharge, exploratory analyses**

|  | H group^#^ | | | HW group^#^ | | | ICU group^#^ | | |  |
| --- | --- | --- | --- | --- | --- | --- | --- | --- | --- | --- |
|  | Twelve-months | Fifteen-months | Eighteen-months | Twelve-months | Fifteen-months | Eighteen-months | Twelve-months | Fifteen-months | Eighteen-months | Sig. |
| Participants n | **44** | **39** | **35** | **60** | **40** | **36** | **44** | **38** | **33** |  |
| Primary outcome |  |  |  |  |  |  |  |  |  |  |
| LPA (min·day^-1^) | 293±21 | 280±19 | 285±19 | 254±22 | 264±19 | 289±20 | 245±24 | 224±20 | 260±21 | p=0.51 |
| MVPA (min·day^-1^) | 10±2 | 6±1 | 8±1 | 12±2 | 9±1 | 6±1† | 14±2 | 9±1 | 7±1† | p=0.06 |
| Steps·day^-1^ | 5835±468 | 4789±399 | 4826±419 | 4364±492 | 4108±409 | 4047±446 | 5065±551 | 4102±431 | 3798±485 | p=0.68 |
| Steps·day^-1^≤5000 | 15(34) | 17(44) | 23(66)† | 21(35) | 22(55) | 20(56) | 14(32) | 23(61) | 16(49) | p=0.27 |
| Sedentary time (min·day^-1^) | 395±23 | 379±18 | 354±20 | 450±24 | 406±18 | 412±21 | 444±27 | 376±20† | 387±23 | p=0.52 |
| Secondary outcomes |  |  |  |  |  |  |  |  |  |  |
| 6MWD (m) | 627±20 | 655±20† | 661±22† | 527±17 | 539±18 | 538±20 | 528±20 | 546±20 | 543±22 | p=0.45 |
| 6MWD≤70%predicted | 4(9) | 2(5) | 1(3) | 13(22) | 8(20) | 5(14) | 13(30) | 9(24) | 6(18) | p=0.52 |
| 1minSTS (reps) | 29±1 | 30±1 | 32±2† | 23±1 | 24±1 | 25±1 | 24±1 | 25±1 | 27±2† | p=0.71 |
| 1minSTS≤70%predicted | 14(32) | 8(21) | 8(23) | 18(30) | 12(30) | 12(33)† | 12(27) | 9(24) | 8(24) | p<0.01* |
| QMS (kgf) | 17±1 | 18±1 | 19±1 | 17±1 | 16±1 | 17±1 | 16±1 | 16±1 | 17±1 | p=0.44 |
| QMS≤70%predicted | 8(18) | 5(13) | 5(14) | 9(15) | 8(20) | 8(22) | 9(21) | 6(16) | 7(21) | p=0.17 |
| Dyspnoea mMRC | 0(0;1) | 1(0;1) | 1(0;1) | 1(0;1) | 1(0;1) | 1(0;1) | 1(0;1) | 1(0;1) | 1(0;1) | p=0.64 |
| mMRC≥2 | 3(7) | 2(5) | 4(11) | 8(13) | 8(20) | 7(19) | 7(16) | 4(11) | 6(18) | p=0.57 |
| Fatigue FACIT-FS | 41(35;49) | 42(31;48) | 45(34;50) | 42(33;50) | 43(32;48) | 43(32;51) | 42(32;48) | 44(36;50) | 43(37;50) | p=0.26 |
| FACIT-FS≤43 | 25(57) | 20(51) | 16(46) | 34(57) | 21(53) | 19(53) | 25(57) | 19(50) | 17(52) | p=0.90 |
| Anxiety HADS-A | 6(3;9) | 6(3;9) | 6(3;9) | 4(2;6) | 5(2;7) | 5(2;8) | 4(2;7) | 3(1;6) | 3(2;5) | p=0.03 |
| HADS-A≥8 | 17(39) | 19(49) | 16(46) | 13(22) | 11(28) | 16(44) | 11(25) | 7(18) | 5(15) | p=0.01* |
| Depression HADS-D | 4(3;6) | 3(1;6) | 3(1;6) | 4(3;6) | 4(2;6) | 4(2;7) | 3(2;6) | 3(1;6) | 3(1;5) | p=0.51 |
| HADS-D≥8 | 9(21) | 8(21) | 5(14) | 12(20) | 9(23) | 10(28) | 8(18) | 6(16) | 4(12) | p=0.23 |
| EQ-5D-5L | 85(75;90) | 80(70;85) | 80(75;90) | 75(65;85) | 80(55;82) | 75(60;85) | 80(65;90) | 80(69;90) | 80(70;90) | p=0.42 |
| EQ-5D-5L≤74/female or 78/male | 11(25) | 14(36) | 8(23) | 32(53) | 16(40) | 18(50) | 17(39) | 15(40) | 12(36) | p=0.08 |
| Data expressed as n, mean±SE, n(%) or median(Q1-Q3). All data are adjusted for age and sex.  Abbreviations. 1minSTS: 1-min sit-to-stand test; 6MWD: 6-min walking distance; EQ-5D-5L: European quality of life - 5 dimensions - 5 levels; FACIT-FS: functional assessment of chronic illness therapy - fatigue; H: home; HADS: hospital anxiety and depression scale; HW, hospital ward; ICU: intensive care unit; LPA: time spent in light physical activity; mMRC: modified Medical Research Council; MVPA: time spent in moderate-to-vigorous physical activity; QMS: quadriceps muscle strength.  *Statistically significant p-value <0.017 among groups, as follows. At twelve-months: 6MWD: p<0.001 between H and HW groups, H and ICU groups; 1minSTS: p=0.003 between H and HW groups, p=0.025 between H and ICU groups; EQ-5D-5L≤74/female or 78/male: p=0.016 between H and HW groups. At fifteen-months: 6MWD: p<0.001 between H and HW groups, H and ICU groups; 1minSTS: p=0.005 between H and HW groups, p=0.04 between H and ICU groups; QMS≤70%predicted: p=0.040 between H and HW groups; HADS-A≥8: p=0.014 between H and HW groups, H and ICU groups. At eighteen-months: 6MWD: p<0.001 between H and HW groups, H and ICU groups; 1minSTS: p=0.003 between H and HW groups; HADS-A≥8: p=0.010 between H and ICU groups, HW and ICU groups; EQ-5D-5L≤74/female or 78/male: p=0.001 between H and HW groups.  †Statistically significant p-value <0.05 within each group, as follows. In H group: Steps·day^-1^≤5000: p=0.003 between twelve and eighteen months; 6MWD: p=0.002 between twelve and fifteen-months, twelve and eighteen-months; 1minSTS: p=0.002 between twelve and eighteen-months. In HW group: MVPA: p=0.007 between twelve and eighteen-months; 1minSTS≤70%predicted: p=0.015 between twelve and eighteen-months. In ICU group: MVPA: p=0.003 between twelve and eighteen-months; Sedentary time: p=0.037 between twelve and fifteen-months; 1minSTS: p=0.005 between twelve and eighteen-months.  ^#^Missing values, as follows. In H group: At twelve: LPA (n=4), MVPA (n=5), Steps·day^-1^ (n=6), Sedentary time (n=6); At fifteen-months: LPA (n=6), MVPA (n=5), Steps·day^-1^ (n=5), Sedentary time (n=5); At eighteen-months: LPA (n=1), MVPA (n=1), Steps·day^-1^ (n=2), Sedentary time (n=2); In HW group: At twelve: LPA (n=26), MVPA (n=27), Steps·day^-1^ (n=26), Sedentary time (n=26), 6MWD, 1minSTS,QMS, Dyspnoea mMRC, FACIT-FS, HADS-A, HADS-D, EQ-5D-5L (n=1); At fifteen-months: Sedentary time (n=6), LPA (n=7), MVPA (n=6), Steps·day^-1^ (n=6); At eighteen-months: Sedentary time (n=6), LPA (n=6), MVPA (n=6), Steps·day^-1^ (n=7); In ICU group: At twelve: Sedentary time (n=17), LPA (n=16), MVPA (n=17), Steps·day^-1^ (n=18); At fifteen-months: Sedentary time (n=8), LPA (n=6), MVPA (n=8), Steps·day^-1^ (n=8); At eighteen-months: Sedentary time (n=10), LPA (n=9), MVPA (n=9), Steps·day^-1^ (n=10). | | | | | | | | | | |

## **Results – Figures**

**a)b)**

**c)d)**

### **Figure S1.** **Physical activity and sedentary behaviour changes of people after COVID-19, during six-month follow-up, starting from twelve-months post-discharge, exploratory analyses**

a) LPA: H group: -13[-64;39]min·day^-1^, p=1.00;-8[-60;44]min·day^-1^, p=1.00; HW group: +11[-44;65]min·day^-1^, p=1.00; +35[-21;92]min·day^-1^, p=0.39; ICU group: -21[-80;39]min·day^-1^, p=1.00; +15[-48;77] min·day^-1^, p=1.00.

b) MVPA: H group: -4[-9;2]min·day^-1^, p=0.30; -2[-7;3]min·day^-1^, p=0.88; HW group: -3[-8;1]min·day^-1^, p=0.25; -5[-9;-1]min·day^-1^, p<0.01†; ICU group: -5[-10;1]min·day^-1^, p=0.11; -7[-12;-2]min·day^-1^, p<0.01†.

c) Steps·day^-1^: H group: -1046[-2121;28], p=0.06; -1009[-2201;182], p=0.13; HW group: -256[-1378;866], p=1.00; -317[-1589;955], p=1.00; ICU group: -962[-2242;317], p=0.21; -1267[-2718;184], p=0.11.

d) Sedentary time: H group: -17[-72;38]min·day^-1^, p=1.00; -42[-101;18]min·day^-1^, p=0.27; HW group: -48[-103;13]min·day^-1^, p=0.19; -39[-102;24]min·day^-1^, p=0.41; ICU group: -68[-133;-3]min·day^-1^, p=0.04†; -57[-128;15]min·day^-1^, p=0.18.

Changes from twelve months are expressed as mean difference[95%CI].

†Statistically significant p-value <0.05 within group., *Statistically significant p-value<0.017 between groups

Abbreviations. COVID-19: Coronavirus Disease 2019; H: home; HW: hospital ward; ICU: intensive care unit; LPA: time spent in light physical activity; MVPA: time spent in moderate-to-vigorous physical activity.
